# Field-Based Affinity Optimization of a Novel Azabicyclohexane Scaffold HIV-1 Entry Inhibitor

**DOI:** 10.3390/molecules24081581

**Published:** 2019-04-22

**Authors:** Megan E. Meuser, Adel A. Rashad, Gabriel Ozorowski, Alexej Dick, Andrew B. Ward, Simon Cocklin

**Affiliations:** 1 Department of Biochemistry & Molecular Biology, Drexel University College of Medicine, Rooms 10307, 10309, and 10315, 245 North 15th Street, Philadelphia, PA 19102, USA; mem484@drexel.edu (M.E.M.); aaa396@drexel.edu (A.A.R.); ad3474@drexel.edu (A.D.); 2 Department of Integrative Structural and Computational Biology, Collaboration for AIDS Vaccine Discovery, The Scripps Research Institute, La Jolla, CA 92037, USA; gozorows@scripps.edu (G.O.); andrew@scripps.edu (A.B.W.)

**Keywords:** bioisosteres, HIV-1 Env, antiviral, surface plasmon resonance, computer-aided drug design

## Abstract

Small-molecule HIV-1 entry inhibitors are an extremely attractive therapeutic modality. We have previously demonstrated that the entry inhibitor class can be optimized by using computational means to identify and extend the chemotypes available. Here we demonstrate unique and differential effects of previously published antiviral compounds on the gross structure of the HIV-1 Env complex, with an azabicyclohexane scaffolded inhibitor having a positive effect on glycoprotein thermostability. We demonstrate that modification of the methyltriazole-azaindole headgroup of these entry inhibitors directly effects the potency of the compounds, and substitution of the methyltriazole with an amine-oxadiazole increases the affinity of the compound 1000-fold over parental by improving the on-rate kinetic parameter. These findings support the continuing exploration of compounds that shift the conformational equilibrium of HIV-1 Env as a novel strategy to improve future inhibitor and vaccine design efforts.

## 1. Introduction

The HIV-1 Env complex, the sole viral protein on the outer surface of the virion, is the main focus of antigen design for antibody-based vaccines and small molecule entry inhibitors [1]. The Env complex itself is a trimer of heterodimeric subunits, gp120 and gp41, and orchestrates the series of events that allow deposition of the viral contents into the host cell, making it a primary determinant of viral infectivity. Over recent years, several structures of recombinant Env trimers have been determined by cryo-electron microscopy and x-ray crystallography, including membrane-extracted trimers and engineered soluble trimer mimics [2,3,4,5,6,7,8,9,10,11]. These structures have revealed many insights about the structural features that govern sensitivity and resistance to neutralization of HIV-1 by antibodies and small molecules.

A number of academic groups and pharmaceutical companies are investing time and effort into the development of small molecule inhibitors of the HIV-1 entry process. Encouraging results have been obtained in one entry inhibitor group, originally developed by Bristol-Myers Squibb, and currently being further developed by ViiV Healthcare [12]. Phase III trials of BMS-663068 (or fostemsavir as it has been dubbed) are ongoing. However, all results from these studies on BMS-663068 prodrug entry inhibitor indicate that it would only be of utility as salvage therapy for highly treatment-experienced patients.

Given the enormous potential of HIV-1 entry inhibitors as both pre- and post-exposure prophylactic regimens, our group has been exploring the modification of piperazine scaffold entry inhibitors [12,13,14,15,16,17,18,19]. We have successfully expanded, designed and tested a number of scaffolds other than piperazine, developing compounds with nanomolar potency and specificity to HIV-1 Env [16,20,21]. Moreover, we have recently demonstrated by using a novel surface plasmon resonance (SPR) interaction assay, that the off-rate of the compounds for a soluble recombinant Env trimer is strongly correlated with the potency of the compounds in the single round infection assay [20]. The results presented herein further extends our work developing novel entry inhibitors by demonstrating the conformational effects of previously disclosed entry inhibitors on the Env complex. Moreover, we demonstrate the affinity optimization of one of these scaffolds, the azabicyclohexane scaffold, which has the most pronounced effect on the conformation of the Env target. The results from this work have broad implications for future HIV-1 entry inhibitor designs that capitalize on the malleability and metastability of the Env protein complex. 

## 2. Results and Discussion

**Figure 1:**
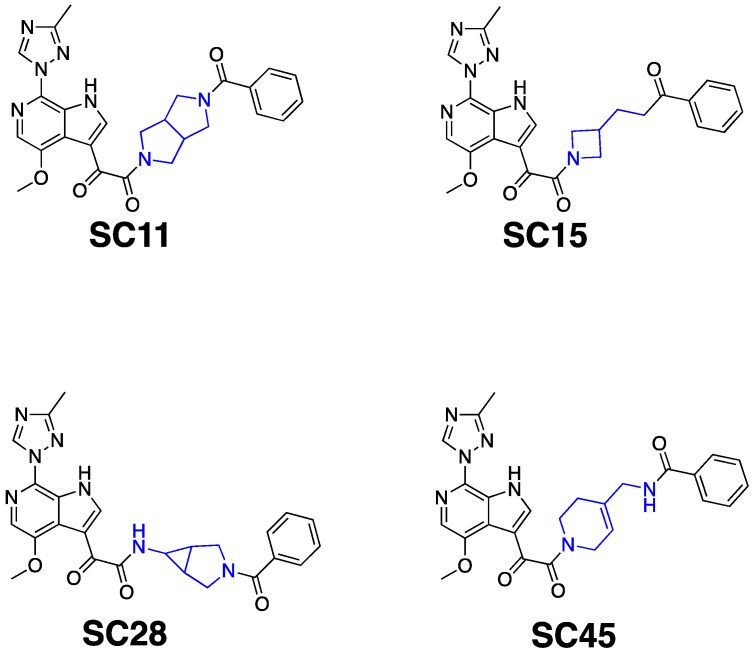
Chemical structures of compounds **SC11**, **SC15**, **SC28**, and **SC45**. The different core scaffolds in each compound are colored blue.

**Figure 2:**
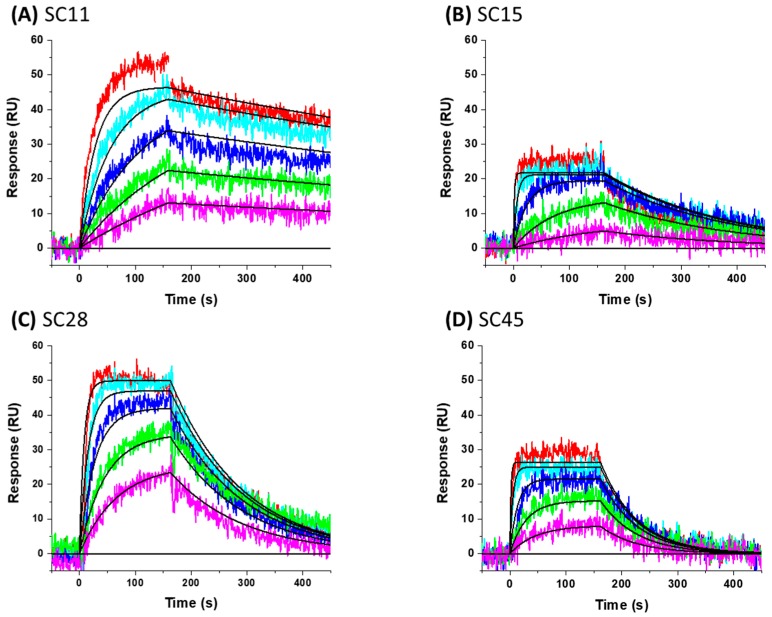
Sensorgrams showing the interaction of small molecule compounds with B41 SOSIP.664 gp140 trimer immobilized on the sensor chip. (**A**) **SC11** (concentrations shown are 10 µM and down in a 1:2 dilution series), (**B**) **SC15** (2 µM, 1:4 dilution series), (**C**) **SC28** (10 µM, 1:2 dilution series), and (**D**) **SC45** (40 µM, 1:3 dilution series). Colored lines represent actual data collected from the dilution series, whereas black lines signify the fits to a 1:1 binding model. Interaction parameters derived from 5 sets of data are given in Table 1.

To gain insight into optimization strategies and mechanisms of action of a selection of our entry inhibitor compounds with differing core scaffolds, we wished to perform molecular docking against the published structure of the HIV-1 SOSIP.664 gp140 trimer. First, however, we had to confirm that the compounds actually do directly interact with the Env complex. We have previously demonstrated the interaction of four of our HIV-1 entry inhibitors with the B41 SOSIP.664 gp140 trimer using surface plasmon resonance [20]. Therefore, as a precursor to molecular modeling, we performed SPR interaction analysis upon **SC11** (dipyrrolidine scaffold), **SC15** (azetidine scaffold), **SC28** (azabicyclo-hexane scaffold), and **SC45** (tetrahydropyridine scaffold) (Figure 1). Figure 2 shows the representative sensograms for each compound interacting with the B41 SOSIP.664 gp140 trimer and Table 1 shows the kinetic parameters obtained after analysis.

**Figure 3:**
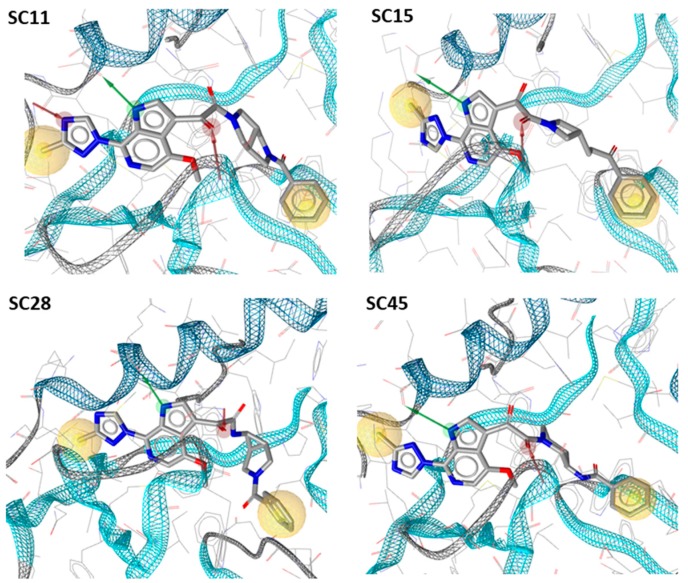
Docking models of compounds **SC11**, **SC15**, **SC28** and **SC45** with B41 SOSIP structure PDB 6MUG.

**Figure 4:**
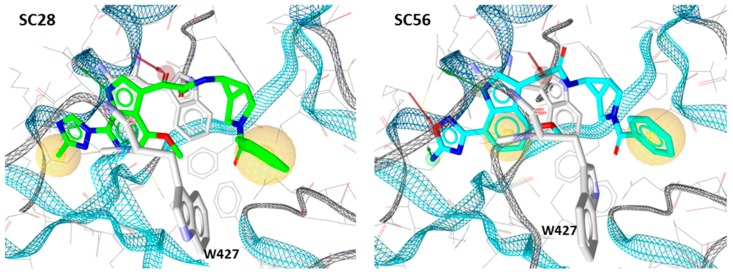
Comparison of the docking models of compounds **SC28** (parental; methyltriazole) and **SC56** (analogue; amine-oxadiazole) highlighting the difference in the orientation of the W427 indole ring.

As predicted, each of the compounds interacted robustly with the B41 SOSIP.664 gp140 trimer, albeit with differing kinetics, providing a clear basis for computational docking of the inhibitors into the structure of soluble Env trimer. Therefore, we docked all of the compounds onto the recently published B41 SOSIP Env structure in complex with BMS-386150, using the binding pocket of the co-crystalized ligand (PDB code: 6MUG) [22] to examine potential differences in binding orientation. Interestingly, compounds **SC11**, **SC15** and **SC45** were easily docked onto the binding site of B41.SOSIP structure, similar to the co-crystalized ligand BMS-386150 (Figure 3), without any need to perform flexible docking. However, the azabicyclo-hexane compound **SC28** did not successfully dock using the previous rigid docking protocol, and instead, we had to use flexible protein-ligand docking in order to achieve a plausible model of **SC28** at the binding site. This need for flexibility in the docking protocol was subsequently found to be due to the fact that the presence of the azabicyclo-hexane moiety, in order to dock to the binding pocket, appears to induce changes in the orientations of certain pocket side chains, especially in the β_20/21_ loop residue W427, and the α1 helix residue W112 (Figure 3 and Figure 4). It is well documented that changes or interactions at one site can have profound effects upon the overall structure and conformation of the Env complex. Moreover, it has been demonstrated recently that the β_20/21_ loop is a regulator of conformational transitions in HIV-1 Env and it is possible that modulation of this region by **SC28** could induce gross changes in structure [23,24].

**Figure 5:**
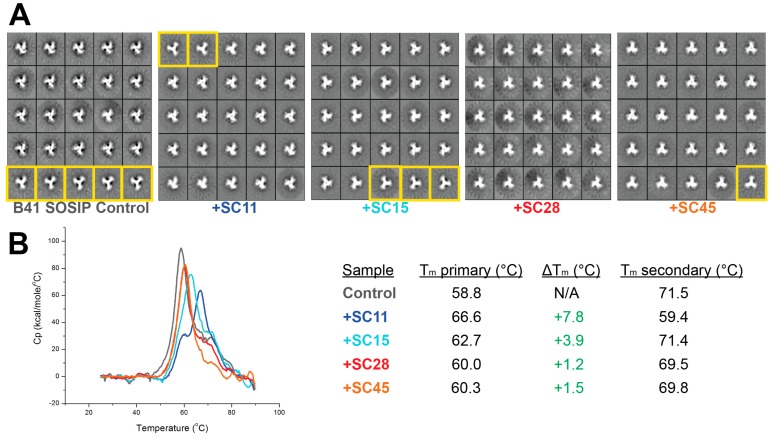
Biophysical characterization of B41 SOSIP.664 incubated with **SC11, SC15, SC28** or **SC45**. (**A**) Representative negative-stain EM 2D class averages. The control sample contained no compound. Averaged particles with phenotypes of “breathing” trimers are highlighted with yellow boxes. All samples were stained with 2% (*w/v*) uranyl formate. (**B**) Differential scanning calorimetry curves (left) and summary of melting temperatures (T_m_) (right). Primary and secondary peaks are those with the highest and second highest intensity (C_p_, specific heat capacity), respectively. ΔT_m_ is the relative change from the inhibitor-free control.

Based on the SPR and docking results, and specifically the participation of the β_20/21_ loop in the binding of **SC28**, we decided to test the effects of four of the compounds on the overall structure of B41 SOSIP.664 using negative-stain electron microscopy (NS-EM). B41 SOSIP.664 has been reported to be a strong immunogen like the “gold standard” BG505 SOSIP.664, with most broadly neutralizing epitopes presented on its surface, yet its appearance by NS-EM suggests a much greater degree of movement of gp120 subunits relative to the three-fold symmetry axis, a behavior sometimes referred to as trimer “breathing” [25,26,27]. When no compound is included, a small population (about 20–25%) of imaged B41 trimers adopted what we infer to be a more open conformation, while the remaining trimers had a more tightly-packed, closed phenotype (Figure 5A). The inclusion of a 10-fold molar excess of any of the four compounds appears to drive the equilibrium from open to closed trimers, at least qualitatively. Statistics of NS-EM analysis are summarized in Table S1. To make a better assessment on the stabilizing effects of the small molecules on the trimer, we used differential scanning calorimetry (DSC) to measure changes in thermostability upon the addition of small molecules (Figure 5B). All four compounds had a positive impact on B41 SOSIP.664 trimer thermostability, with **SC11** having the largest effect (Figure 5B). The combined observations of a measured increase in thermostability (DSC) and an inferred shift towards a more compact state (EM) suggested that the compounds are able to halt the natural dynamics of an Env trimer (that are critical for the virus during receptor recognition and host cell fusion), and are in agreement with the role of the β_20/21_ loop in stabilization of the ground state of Env [23]. Because **SC28** had both experimentally-determined stabilizing effects and in silico suggestions of inducing conformational changes, the results prompted us to search for and design analogues of the azabicyclohexane scaffold with improved affinity. 

In the absence of any experimentally-derived structural information on the bioactive conformation of our inhibitors, we used the docking model of **SC28** as described above as an input into Spark (Cresset, UK) to identify nonclassical bioisosteres of the selected region. From the literature available on the piperazine-based entry inhibitors, it is clear that a primary determinant of potency is the methyltriazole-azaindole head group of the compounds [28,29,30,31]. We, therefore, focused on this region, looking for changes suggested by Spark that may modulate either drug-like parameters or potency, and were significantly different from the methyltriazole-azaindole head group of **SC28**. [16,20,21,32] From this analysis, we docked five compounds onto the B41 SOSIP Env structure (PDB code: 6MUG) [22] using Glide (XP-mode), and looked at the overall ligand-protein interaction energies. Table 2 shows the calculated ligand-protein interaction energies for these compounds in comparison to the parental compound **SC28**. 

Interestingly, all of the azabicyclohexane analogues produced broadly similar overall poses with the distortion of the β_20/21_ loop region but a range of calculated interaction energies. The two compounds with the greatest interaction energies in this analysis were **SC28** and **SC56**, which differ only in the replacement of the methyltriazole with an amine-oxadiazole. This change only appeared to have one large effect upon the binding pocket, with the orientation of the W427 side chain being completely different between the two binding models (Figure 4). This analysis suggests that **SC56** should have greater affinity for the HIV-1 Env than the parental compound, whereas the other modifications run a range of having potentially lower or equal affinities to **SC28**. We therefore chose to synthesize these compounds in order to test the predictions from the design and docking analyses. 

**Figure 6:**
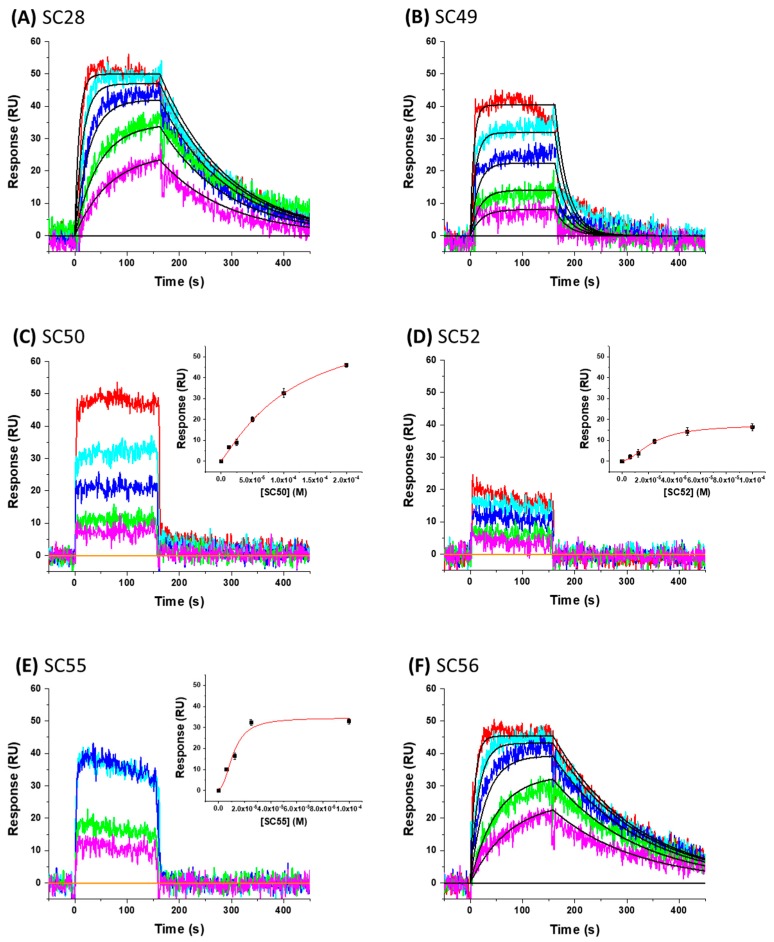
Sensorgrams showing the interaction of small molecule compounds with B41 SOSIP.664 gp140 trimer immobilized on the sensor chip. (**A**) **SC28** (concentrations shown are 10 µM and down in a 1:2 dilution series), (**B**) **SC49** (100 µM, 1:2 dilution series), (**C**) **SC50** (200 µM, 1:2 dilution series), (**D**) **SC52** (100 µM, 1:2 dilution series), (**E**) **SC55** (100 µM, 1:2 dilution series), and (**F**) **SC56** (10 nM, 1:2 dilution series). Colored lines represent actual data, whereas black lines indicate the fits to a 1:1 interaction model. Interaction parameters derived from five sets of data are given in Table 2.

The docking of the new compounds (designated **SC49**, **SC50**, **SC52**, **SC55**, and **SC56**) onto the B41 SOSIP Env structure implies a rank order of affinities. Therefore, after synthesis, and prior to antiviral testing, we sought to establish that the new compounds retained the target specificity of the parental **SC28** compound and whether this rank order held true. We again chose to demonstrate this via SPR. Figure 6 shows the representative sensorgrams for each compound interacting with the B41 SOSIP.664 gp140 trimer and Table 3 shows the kinetic parameters obtained after analysis.

As can be seen in Table 3, all analogues retained target specificity and interacted with the B41 SOSIP.664 gp140 trimer. A range of affinities were observed between the analogues with a general agreement with the docking results, i.e., **SC49** and **SC52** having lower affinities relative to **SC28**. Rather satisfyingly, however, was the finding that **SC56** indeed had a greater affinity for the B41 SOSIP.664 gp140 trimer than **SC28** with a 1000-fold difference improvement in the K_D_ of **SC56** (0.5 nM) in comparison to the parental **SC28** (0.5 µM) and similar overall binding responses to SC28 but over a concentration series 1000-fold more dilute. The dissociation rates of **SC56** and **SC28** are nearly identical indicating that the contributing factor in the kinetics to this increase in affinity is the association rate with the ka parameter of **SC56** increasing by a factor of 1000 in comparison to **SC28**.

After demonstrating that each of the five **SC28** derivatives and **SC28** interact with the B41 SOSIP.664 gp140 trimer, and that we had greatly improved the affinity in one of the analogues, **SC56**, we then tested them for potency against HIV-1 using the HIV-1 single round infection assay. In this system, 293T cells are co-transfected with the envelope-deficient HIV-1 NL4-3 vector (pNL4-3-LucR^+^E^−^; a gift of N. R. Landau, New York University), [33] which carries the luciferase-reporter gene; and the envelope expressing vector from the B41 [26], HxBc2 [34], JR-CSF [35,36], JR-FL [37], or YU-2 [38,39] HIV-1 isolates. This co-transfection yields recombinant single-round infectious envelope-pseudotyped luciferase-reporter HIV-1 viruses. The B41, JR-CSF, JR-FL, and YU-2 envelopes were all originally isolated by directly cloning samples from HIV-1 infected patients and therefore were never subjected to the potential selection imposed by passage of the virus in tissue culture. B41, JR-CSF, JR-FL, and YU-2 are all relatively resistant to neutralization by soluble CD4 and antibodies directed against the HIV-1 envelope glycoproteins and are classified as tier 2 isolates that utilize the CCR5-receptor for entry. Therefore they are representative of the clinically most abundant viruses. The use of the HxBc2 reference strain Env, along with the Envs from four primary R5-tropic viruses allows an assessment of the generality of results obtained. The pseudotyped viruses are then used to infect U87.CD4.CCR5 (B41, JR-CSF, JR-FL, and YU-2) or U87.CD4.CXCR4 (HxBc2) target cells in the presence and absence of compounds and infectivity is quantified by measuring luciferase levels in cell lysates (Luciferase Assay System, Promega, Fitchburg, WI, USA) using a microplate luminometer (GloMax, Promega). The toxicity of the compounds was also assessed in parallel as outlined in Materials & Methods. The results of this analysis are shown in Figure S1 and S2 and the values are summarized in Table 4.

As summarized in Table 4, **SC28** and each of the analogues tested exhibited antiviral effects in the single-round infection assay utilizing viruses pseudotyped with HIV-1 Env from either the B41, HxBc2, JR-CSF, JR-FL, or YU-2 isolates. The analogues exhibited a range of potencies, largely in correlation with the docking and the SPR results. However, we found that **SC56** despite having a much higher affinity for the B41 SOSIP.664 gp140 trimer than **SC28**, had a median IC_50_ only two-fold over that of **SC28.** We have previously demonstrated that the potency of our entry inhibitors has a direct correlation with the kinetic off-rate parameter [20]. Comparison of the kinetics of **SC28** and **SC56** shows that the off-rates for their interaction with the B41 SOSIP.664 gp140 trimer are almost identical, providing a rationale for their similar potencies, and further corroborating our previous study. 

## 3. Material and Methods

### 3.1. General Information

DMEM, FBS, penicillin, streptomycin and L-glutamine, G418, puromycin, Multiskan™ GO Microplate Spectrophotometer, FEI Talos Arctica electron microscope, FEI Ceta 16M CMOS camera (Thermo Scientific, Waltham, MA, USA); BL21-Codon Plus (DE3)-RIL Competent Cells (Agilent Technologies, Wilmington, DE, USA); Talon cobalt resin affinity column (Clonetech Laboratories, Mountain View, CA, USA); calcium phosphate, 5X luciferase lysis buffer, luciferase assay substrate, GloMax 96 microplate luminometer (Promega, Madison, WI, USA); mouse anti-p24 (ab9071, Abcam, Cambridge, MA, USA); Triton X-100, O-phenylenediamine, phosphate-citrate buffer with sodium perborate, DMSO (Sigma-Aldrich, St. Louis, MO, USA); 96-well luminometer-compatible tissue culture plates (Greiner Bio-one, Monroe, NC, USA); Cell Counting Kit-8 cell proliferation and cytotoxicity assay (Dojindo Molecular Technologies, Rockville, MD, USA); 96-well tissue culture plates (Olympus Plastics, San Diego, CA, USA); ProteOn XPR36 SPR Protein Interaction Array System, GLH sensorchip, (Bio-Rad Laboratories, Hercules, CA, USA); MicroCal VP-Capillary differential scanning calorimeter, Automated Origin 7.0 software (Malvern Panalytical, Westborough, MA, USA); Protein Preparation Wizard implemented with Maestro (Schrödinger Maestro Version 11.5.011, New York, NY, USA, MM share Version 4.1.011, New York, NY, USA, Release 2018-1, Platform Darwin-x86_64). **SC11** was synthesized as outlined in Tuyishime et al. [21]. **SC15**, **SC28**, and **SC45** were synthesized as outlined in Tuyishime et al. [16]. 

### 3.2. Chemistry 

#### 3.2.1. Synthesis of **SC49**

##### General Procedure for the Preparation of **2**

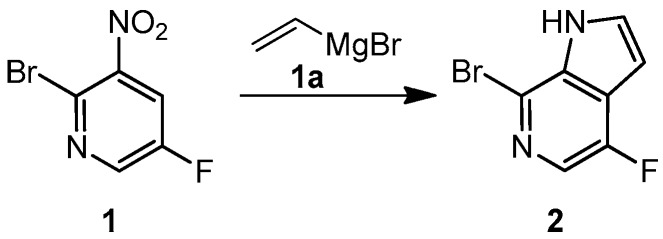



Vinylmagnesium bromide **1a** (1 M, 543.04 mL) was cooled below −60 °C with vigorous stirring under N_2_. A solution of 1 (30 g, 136 mmol) in THF (100 mL) was added dropwise slowly that the temperature was kept below −60 °C. The reaction mixture was warmed to −40 to −50 °C and stirred for an additional 1 h. TLC (petroleum ether: ethyl acetate = 2:1, Rf = 0.47) show that the reaction was complete. Saturated aqueous NH_4_Cl (200 mL) was added slowly. The layers were separated, and the aqueous layer was extracted with EtOAc (3 × 200 mL). The organic extracts were washed with brine (200 mL), dried over Na_2_SO_4_, filtered, and concentrated. To the residue CH_2_Cl_2_ (100 mL) was added and the solid formed was collected by filtration and washed with CH_2_Cl_2_ (50 mL) to give **2** (8 g, 27% yield) as a brown solid. ^1^H-NMR (400 MHz CDCl_3):_ δ 8.61–8.91 (m, 1 H) 7.93 (d, *J* = 1.6 Hz, 1 H) 7.43 (t, *J* = 2.4 Hz, 1 H) 6.78 (t, *J* = 2.4 Hz, 1 H).

##### General Procedure for the Preparation of **3**

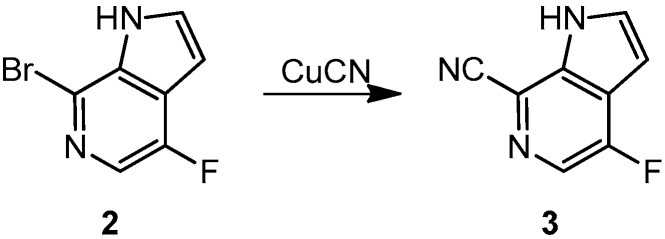



A mixture of **2** (4 g, 18.6 mmol) and CuCN (3.3 g, 37.2 mmol) in DMF (30 mL) was stirred at 150 °C for 1 h, after which TLC (petroleum ether: ethyl acetate = 2: 1, Rf = 0.51) showed reaction completion. The mixture was diluted with EtOAc (100 mL), washed with water (100 mL), brine (100 mL), and concentrated. The residue was purified by column chromatography on silica gel and eluted with petroleum ether: ethyl acetate = 4: 1 to give **3** (900 mg, 30% yield) as a yellow solid. ^1^H-NMR: ET6983-5-P1A (400 MHz DMSO-*d*_6):_ δ 12.75 (br. s., 1 H), 8.27 (d, *J*=1.2 Hz, 1 H), 7.86 (d, *J* = 3.2 Hz, 1 H), 6.83 (d, *J* = 2.8 Hz, 1 H).

##### General Procedure for the Preparation of **4**

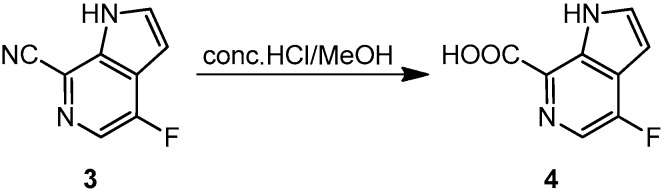



A mixture of **3** (800 mg, 4.96 mmol) in MeOH (10 mL) and conc. HCl (10 mL) was stirred at 90 °C for 16 h. TLC (petroleum ether: ethyl acetate = 2:1, Rf = 0.01) showed that the reaction was complete. The organic solvent was evaporated, and the precipitate was filtered off and dried to give **4** (500 mg, 56% yield) as a brown solid. ^1^H-NMR (400 MHz MeOD): δ 8.39 (d, *J* = 4.0 Hz, 1 H), 8.23 (d, *J* = 2.8 Hz, 1 H), 7.15 (d, *J* = 3.2 Hz, 1 H).

##### General Procedure for the Preparation of **5**

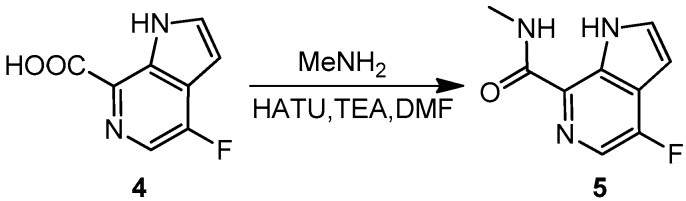



A solution of **4** (450 mg, 2.50 mmol), DIEA (967 mg, 7.49 mmol), and HATU (1.04 g, 2.75 mmol) in THF (10 mL) was stirred at 25 °C for 0.5 h. Then methylamine (675 mg, 9.99 mmol, HCl salt) was added, and the mixture was stirred at 25 °C for 16 h. TLC (dichloromethane: methanol = 20: 1, Rf = 0.65) showed that the reaction was complete. The mixture was diluted with EtOAc (20 mL), washed with water (20 mL), brine (20 mL) and dried over Na_2_SO_4_, and concentrated. The residue was purified by column chromatography on silica gel and eluted with petroleum ether: EtOAc = 5:1 to give **5** (400 mg, 83% yield) as a white solid. ^1^H-NMR: ET6983-9-P1A (400 MHz, CDCl_3_): δ 10.50 (br. s., 1 H), 8.01 (d, *J* = 1.2 Hz, 1 H), 7.92 (br. s., 1 H), 7.47 (br. s., 1 H), 7.26 (s, 1 H), 6.69 (br. s., 2 H), 3.07 (d, *J* = 4.8 Hz, 1 H).

##### General Procedure for the Preparation of **6**

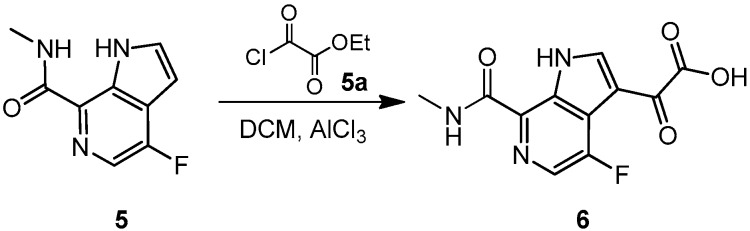



Compound **5** (370 mg, 1.92 mmol) was added to a mixture of AlCl_3_ (1.53 g, 11.5 mmol) and 1-ethyl-3-methylimidazol-3-ium chloride (566 mg, 3.83 mmol). Then, **5a** (523 mg, 3.83 mmol) was added slowly to the solution, and the mixture was stirred at 25 °C for 15 h. TLC (ethyl acetate: petroleum ether = 2:1, Rf = 0.01) showed that the conversion was more than 50% and LCMS showed the desired mass of the product. Water was added (20 mL) slowly to the mixture at 0 °C. The precipitate was filtered off, and dried to give **6** (200 mg, crude) as a yellow solid.

##### General Procedure for the Preparation of **7**

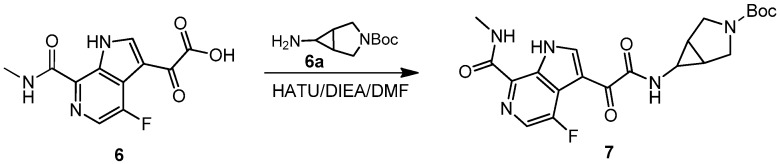



To a solution of **6** (200 mg, 754 mmol and DIEA (292 mg, 2.26 mmol) in DMF (5 mL) was added HATU (287 mg, 754 mmol). After stirred at 25 °C for 30 min, 6a (150 mg, 754 mmol) was added and the mixture was stirred at 25 °C for 16 h. TLC (petroleum ether: ethyl acetate = 0:1, Rf = 0.3) showed that the reaction was complete and LCMS showed the desired mass of the product. The mixture was diluted with EtOAc (20 mL), washed with water (20 mL), brine (20 mL), dried over Na_2_SO_4_, and concentrated to give **7** (300 mg, crude) as a yellow solid.

##### General Procedure for the Preparation of **8**

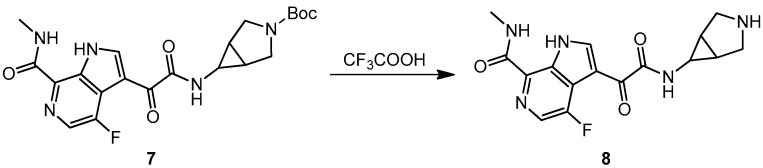



A solution of **7** (300 mg, 674 mmol) in TFA (4 mL) was stirred at 25 °C for 16 h. LCMS showed that the reaction was complete. The mixture was concentrated to give the crude product 8 TFA salt (200 mg, crude) as a yellow solid.

##### General Procedure for the Preparation of **SC49**

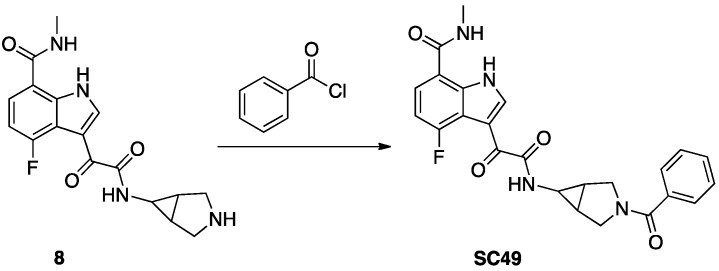



To a solution of **8** (200 mg, 579 mmol) and DIEA (150 mg, 1.16 mmol) in DCM (5 mL) was added 8a (163 mg, 1.16 mmol). Then the mixture was stirred at 25 °C for 16 h. LCMS showed that the desired product was produced. The mixture was concentrated. The residue was purified by neutral prep-HPLC to give **SC49** (22 mg, 8% yield) as a light yellow solid. ^1^H-NMR: ET6983-14-P1B (400 MHz CDCl_3_): δ 11.25 (br. s., 1 H), 9.26 (s, 1 H), 8.20 (d, *J* = 2.4 Hz, 1 H), 7.92 (d, *J* = 4.4 Hz, 1 H), 7.52 (br. s., 1 H), 7.36–7.48 (m, 4 H), 4.34 (d, *J* = 12.0 Hz, 1 H), 3.46–3.90 (m, 3 H), 3.08 (d, *J* = 4.8 Hz, 3 H), 2.61 (br. s., 1 H), 1.75–2.00 (m, 2 H).

#### 3.2.2. The Synthesis of SC50

##### General Procedure for the Preparation of **9**

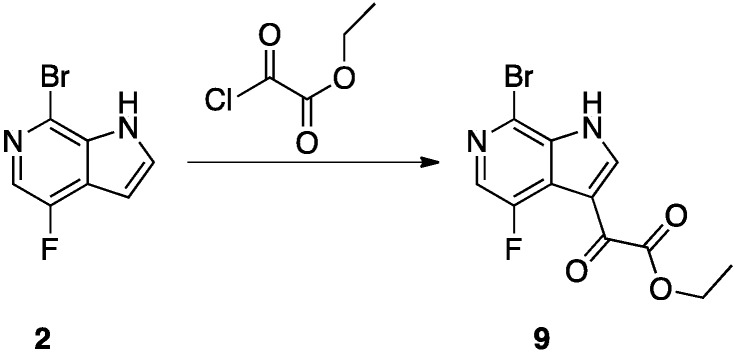



To a mixture of 1-ethyl-3-methylimidazol-3-ium chloride (4.1 g, 27.9 mmol) was added AlCl_3_ (11.2 g, 83.7 mmol) in portions at 0 °C and stirred for 30 min, then 2 (3.0 g, 13.9 mmol) was added in portions at 0 °C under N_2_. The mixture was stirred at 0 °C for 0.5 h, then ethyl 2-chloro-2-oxo-acetate (3.8 g, 27.9 mmol) was added dropwise, then the mixture was stirred at 25 °C for 2 h. TLC (petroleum ether: ethyl acetate = 3: 1, R_f_ = 0.2) showed the reaction was occurred and one new spot was generated. The mixture was poured onto water (50 mL) and stirred for 10 min. The aqueous phase was extracted with ethyl acetate (50 mL × 3 mL). The combined organic phase was washed with brine (30 mL × 2mL), dried over Na_2_SO_4_, filtered and concentrated in vacuum to give **9** (3 g, 68% yield) as a brown solid. ^1^H-NMR: ET6822-13-P1A (400 MHz CDCl_3_): δ 8.56 (s, 1 H), 8.40 (s, 1 H), 7.88 (d, *J* = 2.4 Hz, 1 H), 4.24–4.35 (m, 2 H), 1.23–1.36 (m, 3 H).

##### General Procedure for the Preparation of **10**

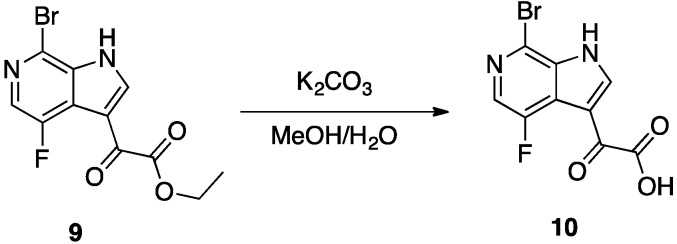



To a mixture of **9** (3 g, 9.5 mmol) in MeOH (30 mL) and H_2_O (10 mL) was added K_2_CO_3_ (2.6 g, 19 mmol) in one portion at 25 °C under N_2_. The mixture was stirred at 25 °C for 5 h. TLC (petroleum ether:ethyl acetate = 1: 1, R_f_ = 0.03) showed the reaction was completed. The mixture was concentrated in reduced pressure at 45 °C. The residue was poured onto water (50 mL) and stirred for 10 min. The mixture was acidified with HCl (1 M) to pH ~4, the aqueous phase was extracted with ethyl acetate (50 mL × 3mL). The combined organic phase was washed with brine (30 mL × 2mL), dried with anhydrous Na_2_SO_4_, filtered and concentrated in vacuum to give **10** (2.0 g, 73% yield) as a yellow solid. ^1^H-NMR: ET6822-14-P1A (400 MHz DMSO-*d*_6_): δ 13.35 (br. s., 1 H), 8.47–8.65 (m, 1 H), 8.12 (d, *J* = 2.4 Hz, 1 H).

##### General Procedure for the Preparation of **11**

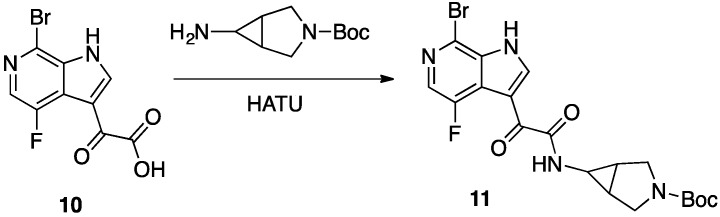



To a mixture of **10** (900 mg, 3.1 mmol) in DMF (30 mL) was added HATU (1.55 g, 4.08 mmol) and DIEA (1.22 g, 9.4 mmol) in one portion at 25 °C under N_2_. The mixture was stirred at 25 °C for 30 min, then 6a (745 mg, 3.7 mmol) was added in portions and the mixture was stirred for 5 h. TLC (petroleum ether:ethyl acetate = 1:1, R_f_ = 0.6.) showed one main spot was generated. The mixture was poured onto water (50 mL) and stirred for 10 min. The aqueous phase was extracted with ethyl acetate (30 mL × 2). The combined organic phase was washed with brine (20 mL × 2), dried with anhydrous Na_2_SO_4_, filtered and concentrated in vacuum. The residue was purified by silica gel chromatography (column height: 250 mm, diameter: 100 mm, 100–200 mesh silica gel, petroleum ether:ethyl acetate = 1:1) to give 11 (2.0 g, 68% yield) as a yellow solid. 

##### General Procedure for the Preparation of **12**

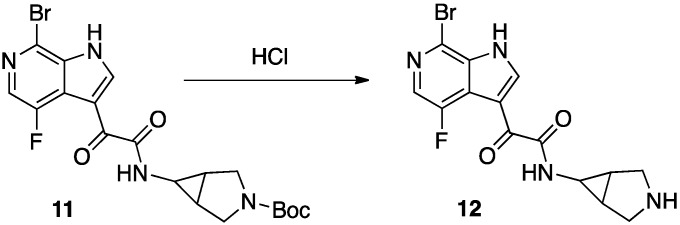



A mixture of **11** (2.0 g, 4.28 mmol) in HCl/EtOAc (20 mL) was stirred at 25 °C for 5 h. LCMS showed the reaction was completed, and the desired product was generated. The mixture was filtered and washed with EtOAc, and concentrated in vacuum to give **12** (1.2 g, 76% yield) as a yellow solid. 

##### General Procedure for the Preparation of **13**

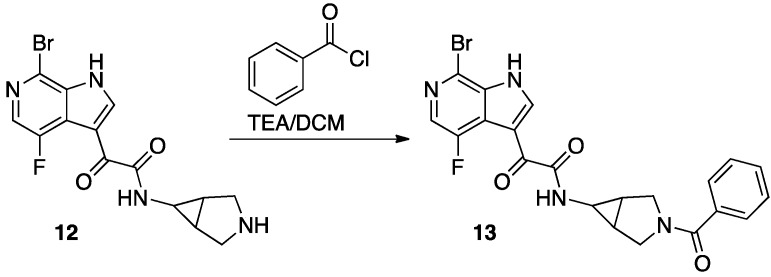



To a mixture of **12** (1.5 g, 4.09 mmol) and benzoyl chloride (1.15 g, 8.17 mmol) in DCM (30 mL) was added TEA (413 mg, 4.09 mmol) in one portion at 25 °C under N_2_. The mixture was stirred at 25 °C for 2 h. LC-MS showed the reaction was completed. The mixture was concentrated in reduced pressure at 45 °C. The residue was purified by prep-HPLC (acid conditions) to give **13** (600 mg, 31% yield) as a yellow solid. ^1^H-NMR (400 MHz DMSO-*d*_6_): δ 13.22 (br. s., 1 H), 9.04 (d, *J* = 4.8 Hz, 1 H), 8.80 (d, *J* = 2.4 Hz, 1 H), 8.10 (d, *J* = 2.8 Hz, 1 H), 7.22–7.56 (m, 5 H), 4.01 (d, *J* = 12.4 Hz, 1 H), 3.67 (d, *J* =7.2 Hz, 1 H), 3.42 (dd, *J* = 18.4, 11.2 Hz, 2 H), 2.51–2.57 (m, 1 H), 1.88 (d, *J* = 7.6 Hz, 2 H).

##### General Procedure for the Preparation of **SC50**

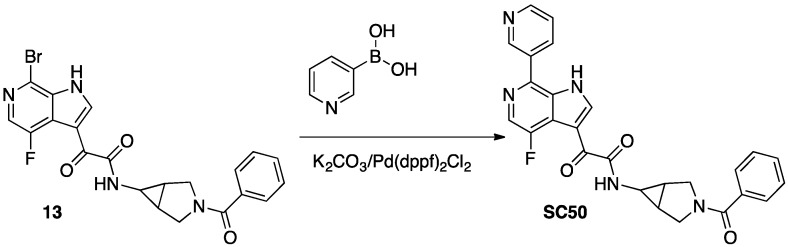



To a solution of **13** (100 mg, 0.212 mmol), 3-pyridylboronic acid (39 mg, 0.318 mmol) in dioxane (5 mL) and H_2_O (1 mL) was added Pd(dppf)Cl_2_ (15.5 mg, 21 mmol) and K_2_CO_3_ (87.9 mg, 637 mmol) in one portion at 25 °C then heated at 110 °C for 12 h. TLC (dichloromethane:methanol = 10:1, R_f_ = 0.4) showed reaction completion, LCMS showed the desired product was formed. After cooling to 25 °C, ethyl acetate (30 mL) was added and combined and concentrated under reduce pressure. The residue was purified by prep-TLC (dichloromethane:methanol = 10:1, R_f_ = 0.4) to give **SC50** (90 mg, 91% yield) as an off-white solid. ^1^H-NMR (400 MHz MeOD): δ 8.96 (d, *J* =1.6 Hz, 1 H), 8.91 (s, 1 H), 8.68 (d, *J* = 4.8 Hz, 1 H), 8.19–8.32 (m, 2 H), 7.64 (dd, *J* = 8.0, 4.8 Hz, 1 H), 7.41–7.51 (m, 5 H), 4.56 (s, 1 H), 4.21 (d, *J* = 12.4 Hz, 1 H), 3.72–3.80 (m, 1 H), 3.57–3.71 (m, 2 H), 2.54 (s, 1 H), 1.88–2.05 (m, 2 H).

#### 3.2.3. Synthesis of **SC52**

##### Preparation of *tert*-Butyl (3-benzoyl-3-azabicyclo [3.1.0]hexan-6-yl) Carbamate (**14**)

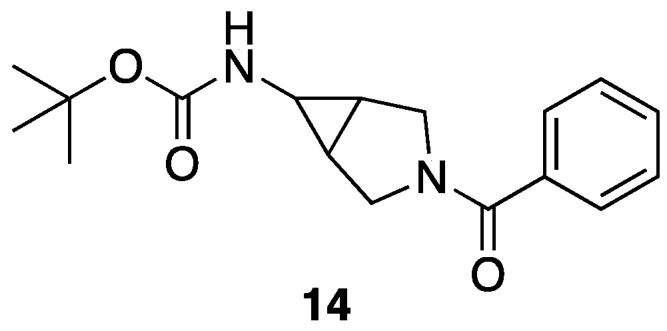



At 0 °C, benzoyl chloride (97.4 mg, 0.70 mmol) was added to a mixture of *tert*-butyl (3-azabicyclo[3.1.0]hexan-6-yl)carbamate (125.0 mg, 0.63 mmol) and TEA (127.0 mg, 1.26 mmol) in DCM (5 mL) dropwise. The resulting mixture was stirred at 0 °C for 30 min. The reaction was then quenched with H_2_O (20 mL). The mixture was extracted with DCM (50 mL × 2). The combined organic layers was washed with brine (100 mL), dried over anhydrous Na_2_SO_4_ and concentrated under reduced pressure. The residue was purified by column chromatography (silica gel, 0–100% EtOAc in petroleum ether) to give the title product (150.0 mg, 78.7% yield) as colorless oil. LC-MS (ESI): *m*/*z* [M + 1]^+^ = 303.14.

##### Preparation of (6-Amino-3-azabicyclo[3.1.0]hexan-3-yl)(phenyl) Methanone (**15**)

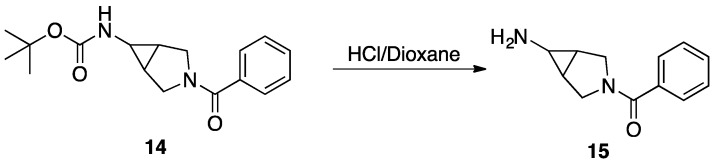



To a solution of **14** (150 mg, 0.496 mmol) in DCM (5 mL), HCI-dioxane (2 M, 3 mL) was added. The resulting mixture was stirred at room temperature for 8 h. After completion of the reaction indicated by LCMS and TLC, the mixture was concentrated under reduced pressure to afford the crude amine **15** (120 mg, 100% yield) as colorless oil, which was used in the next step directly without further purification. LC-MS (ESI): *m*/*z* [M + 1]^+^ = 203.10.

##### Preparation of 6-Allylpyrazolo[1,5-a]pyrimidine-5,7-diol (**17**)

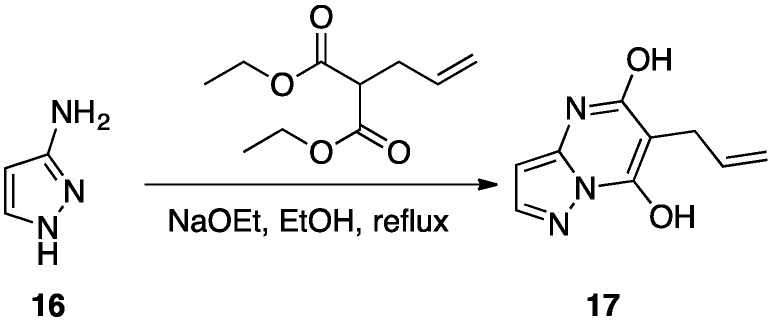



To a solution of 1*H*-pyrazol-3-amine **16** (15.2 g, 182.9 mmol) in EtOH (200 mL) were added NaOEt (24.9 g, 365.8 mmol), followed by diethyl 2-allylmalonate (36.6 g, 182.9 mmol). The resulting mixture was stirred at 100 °C overnight. After cooled down to RT, the precipitate was collected by filtration and dissolved in H_2_O (500 mL). The resulted solution was acidified to pH~2 with 1N HCI, and filtered to give the desired product **17** (22 g, 62.9% yield) as a white solid. LC-MS (ESI): *m*/*z* [M + 1]^+^ = 192.33.

##### Preparation of 6-Allyl-5,7-dichloropyrazolo[1,5-a]pyrimidine (**18**)

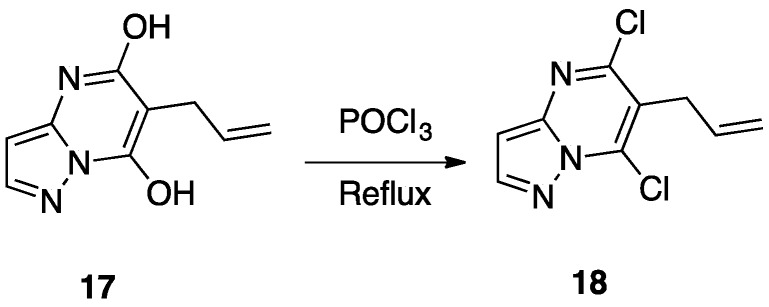



A solution of **17** (16.5 g, 86.4 mmol) in POCI_3_ (100 mL) was stirred at 100 °C overnight. After cooled down to RT, excess POCl_3_ was removed under reduced pressure to afford the **18** (17.2 g, 87.3% yield) as brown oil, which was used in the next step directly without further purification. LC-MS (ESI): *m*/*z* [M + 1]^+^ =228.14, 230.14.

##### Preparation of 6-Allyl-5-chloro-N-(4-methoxybenzyl)pyrazolo[1,5-a] pyrimidin-7-amine (**19**)

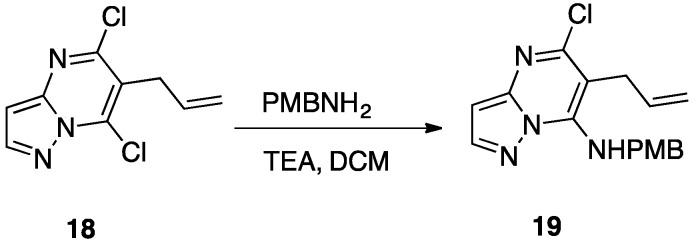



To a solution of **18** (17.2 g, 75.4 mmol) and TEA (15.2 g, 150.8 mmol) in DCM (250 mL), PMBNH_2_ (10.3 g, 75.4 mmol) was added. The mixture was stirred at room temperature for 24 h. The reaction solution was diluted with H_2_O (250 mL), and extracted with DCM (250 mL × 2). The combined organic layers were washed with brine (300 mL), dried over anhydrous Na_2_SO_4_, filtered and concentrated under reduced pressure. The residue was purified by column chromatography (silica gel, 0–50% EtOAc in petroleum ether) to give the **19** (17.4 g, 70.2% yield) as a white solid. LC-MS (ESI): *m*/*z* [M + 1]^+^ = 329.09, 331.04; ^1^H-NMR (400 MHz, DMSO-*d_6_*): *δ* 8.12 (d, *J* = 2.4 Hz, 1H), 8.01 (t, *J* = 6.8 Hz, 1H), 7.21–7.17 (m, 2H), 6.89–6.84 (m, 2H), 6.41 (d, *J* = 2.0 Hz, 1H), 6.00–5.90 (m, 1H), 5.12–5.08 (m, 1H), 4.99 (d, *J* = 6.8 Hz, 2H), 4.90–4.88 (m, 1H), 3.71 (s, 3H), 3.47–3.45 (m, 2H).

##### Preparation of 5-Chloro-8-(4-methoxybenzyl)-8*H*-pyrazolo[1,5-a]pyrrolo[3,2-e]pyrimidine (**20**)

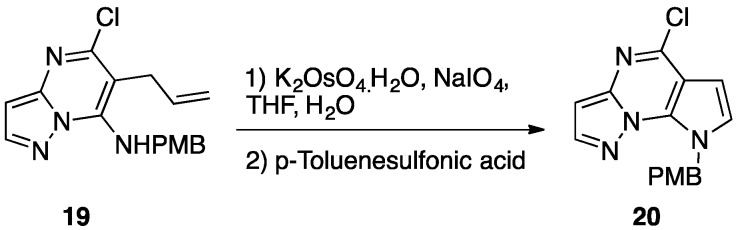



A mixture of **19** (2.2 g, 6.69 mmol), K_2_OsO_4_·H2O (234.8 mg, 0.67 mmol) and NaIO_4_ (5 g, 23.4 mmol) in THF/H_2_O (25 mL, 4:1, *v*/*v*) was stirred at 0 °C for 5 h, and then the PTSA (1.15 g, 6.69 mmol) was added. The mixture was stirred at room temperature for 2 h. The reaction solution was diluted with H_2_O (50 mL), and extracted with ethyl acetate (50 mL × 2). The combined organic layers were washed with brine (100 mL), dried over anhydrous Na_2_SO_4_, filtered and concentrated under reduced pressure. The residue was purified by column chromatography (silica gel, 0–30% EtOAc in petroleum ether) to give **20** (1.1 g, 52.6% yield) as a white solid. LC-MS (ESI): *m/z* [M + 1]^+^ = 313.04, 315.04; ^1^H-NMR (400 MHz, DMSO-*d_6_*): *δ* 8.23 (d, *J* = 2.4 Hz, 1H), 7.41 (d, *J* = 3.2 Hz, 1H), 7.32–7.30 (m, 2H), 6.89–6.86 (m, 2H), 6.73 (d, *J* = 2.4 Hz, 1H), 6.71 (d, *J* = 3.6 Hz, 1H), 5.94 (s, 2H), 3.70 (s, 3H).

##### Preparation of 8-(4-Methoxybenzyl)-5-(3-methyl-1*H*-1,2,4-triazol-1-yl)-8*H*-pyrazolo[1,5-a]-pyrrolo[3,2-e]pyrimidine (**21**)

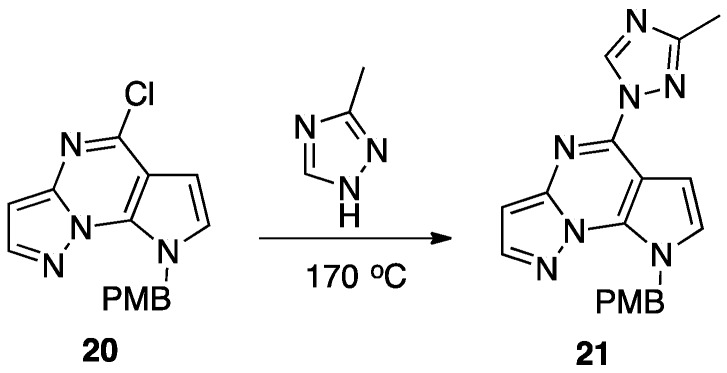



A mixture of **20** (1.1 g, 3.5 mmol) and 3-methyl-1*H*-1,2,4-triazole (1.4 g, 17.5 mmol) was stirred at 170 °C for 2 h. After cooled down to room temperature, the reaction mixture was diluted with H_2_O (30 mL) and extracted with DCM (30 mL × 2). The combined organic phases were washed with brine (30 mL), dried over anhydrous Na_2_SO_4_, filtered and concentrated under reduced pressure. The residue was purified by column chromatography (silica gel, 0–100% EtOAc in petroleum ether) to give **21** (732 mg, 58.2% yield) as a yellow solid. LC-MS (ESI): *m*/*z* [M + 1]^+^ = 360.35.

##### Preparation of 5-(3-Methyl-1*H*-1,2,4-triazol-1-yl)-8*H*-pyrazolo[1,5-a] pyrrolo[3,2-e]-pyrimidine (**22**)

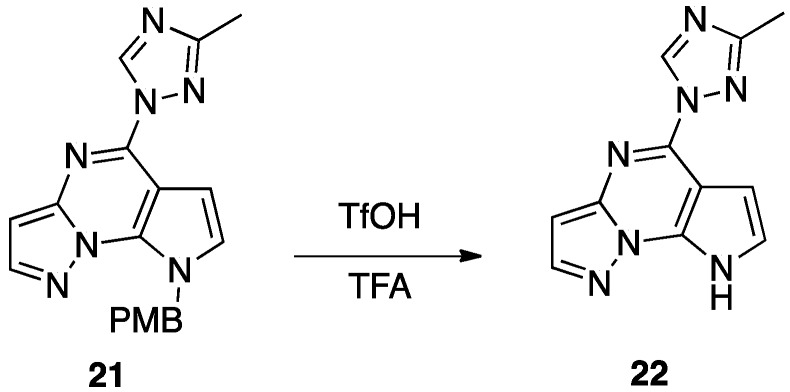



A solution of **21** (732 mg, 2.04 mmol) in TfOH/TFA (7 mL, 2:5, *v*/*v*) was stirred at room temperature for 1h. The reaction mixture was concentrated under reduced pressure. The residue was purified by prep-HPLC (C18, 30–100% acetonitrile in H_2_O with 0.1% formic acid) to give **22** (166 mg, 34% yield) as a white solid. LC-MS (ESI): *m*/*z* [M + 1]^+^ = 240.19; ^1^H-NMR (400 MHz, DMSO-*d_6_*): *δ* 13.37 (br. s, 1H), 9.41 (s, 1H), 8.21 (d, *J* = 2.4 Hz, 1H), 7.30 (d, *J* = 3.2 Hz, 2H), 7.21 (d, *J* = 3.2 Hz, 1H), 6.69 (d, *J* = 2.4 Hz, 1H), 2.47 (s, 3H).

##### Preparation of Ethyl 2-(5-(3-methyl-1*H*-1,2,4-triazol-1-yl)-8*H*-pyrazolo [1,5-a]pyrrolo[3,2-e]-pyrimidin-8-yl)acrylate (**23**)

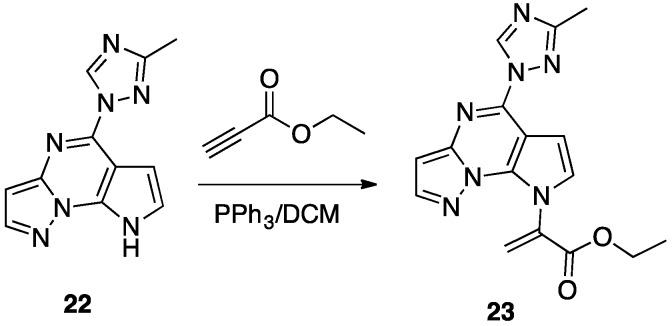



To a stirred solution of **22** (166 mg, 0.693 mmol) and ethyl propiolate (68 mg, 0.693 mmol) in DCM (5 mL) was added the solution of PPh_3_ (181.8 mg, 0.693 mmol) in DCM (3 mL) dropwise at 0 °C. The reaction mixture was stirred at room temperature for 4 h and then concentrated under reduced pressure. The residue was purified by column chromatography (silica gel, 0–100% EtOAc in petroleum spirit) to give **23** (85 mg, 36.4% yield) as a white solid. LC-MS (ESI): *m*/*z* [M + 1]^+^ = 338.10.

##### Preparation of 2-(5-(3-Methyl-1*H*-1,2,4-triazol-1-yl)-8*H*-pyrazolo [1,5-a]pyrrolo[3,2-e]-pyrimidin-8-yl)acrylic acid (**24**)

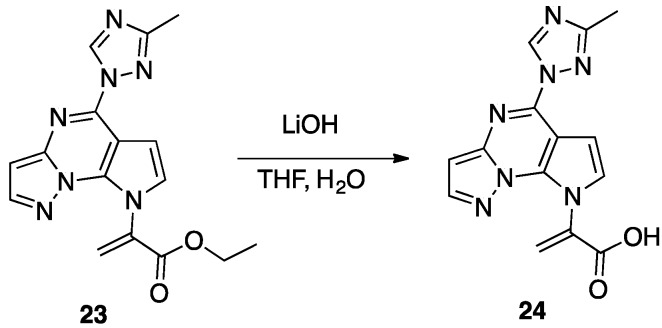



To a solution of **23** (85 mg, 0.252 mmol) in THF(5 mL), LiOH (12.1 mg, 0.504 mmol) dissolved in H_2_O (2 mL) was added. The resulting mixture was stirred at room temperature for 1 h. The reaction mixture was acidified to pH~5 with 1N aqueous HCI solution and then diluted with EtOAc (20 mL) and H_2_O (20 mL), extracted with EtOAc (30 mL × 2). The combined organic phases were dried over anhydrous Na_2_SO_4_ and concentrated under reduced pressure. The residue was purified by column chromategraphy (silica gel, 0–10% MeOH in DCM) to give **24** (35 mg, 45% yield) as a white solid. LC-MS (ESI): *m*/*z* [M + 1]^+^ = 310.09.

##### Preparation of *N*-(3-benzoyl-3-azabicyclo[3.1.0]hexan-6-yl)-2-(5-(3- methyl-1*H*-1,2,4-triazol-1-yl)-8*H*-pyrazolo[1,5-a]pyrrolo[3,2-e]pyrimidin-8-yl)acrylamide (**SC52**)

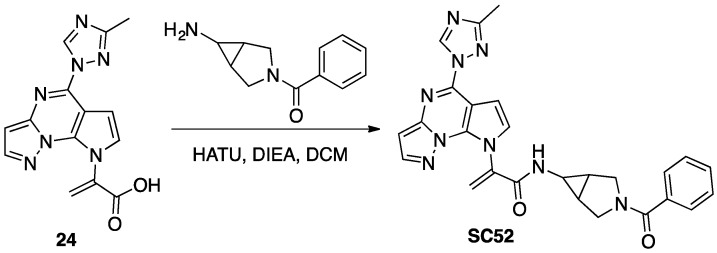



To a mixture of **24** (35 mg, 0.113 mmol), DIPEA (29.2 mg, 0.226 mmol) and (6-amino-3-azabicyclo[3.1.0]hexan-3-yl)(phenyl)methanone (22.9 mg, 0.113 mmol) in DCM (5 mL) was added HATU (51.7 mg, 0.136 mmol) slowly at RT. The reaction mixture was stirred at room temperature for 30 min. After reaction completion, the mixture was diluted with H_2_O (10 mL), and then extracted with DCM (20 mL × 2). The combined organic layers were washed with brine, dried over anhydrous Na_2_SO_4_ and concentrated under reduced pressure. The residue was purified by prep-HPLC (C18 column, 30%~100% MeCN in H_2_O, with 0.1% formic acid in H_2_O) to give the title product (8.5 mg, 15.2% yield) as a white power. LC-MS (ESI): *m*/*z* [M + 1]^+^ = 494.09; ^1^H-NMR (400 MHz, DMSO-*d_6_*): *δ* 9.43 (s, 1H), 8.63 (d, *J* = 3.2 Hz, 1H), 8.12 (d, *J* = 2.4 Hz, 1H), 7.47–7.36 (m, 4H), 7.34–7.28 (m, 2H), 6.68 (d, *J* = 2.4 Hz, 1H), 6.30 (d, *J* = 2.0 Hz, 1H), 6.06 (d, *J* = 2.0 Hz, 1H), 3.95 (d, *J* = 12.4 Hz, 1H), 3.69–3.65 (m, 1H), 3.47–3.43 (m, 1H), 3.35 (d, *J* = 11.2 Hz, 1H), 2.48 (s, 3H), 2.35–2.32 (m, 1H), 2.03–1.97 (m, 1H), 1.82–1.75 (m, 2H).

#### 3.2.4. The Synthesis of **SC55**

Synthesis of 2-(7-(5-amino-1,2,4-oxadiazol-3-yl)-4-fluoro-1*H*-indol-3-yl)-*N*-(3-benzoyl-3-azabicyclo[3.1.0]hexan-6-yl)-2-oxoacetamide (**SC55**)

##### Preparation of 7-Bromo-4-fluoro-1*H*-indole (**26**)

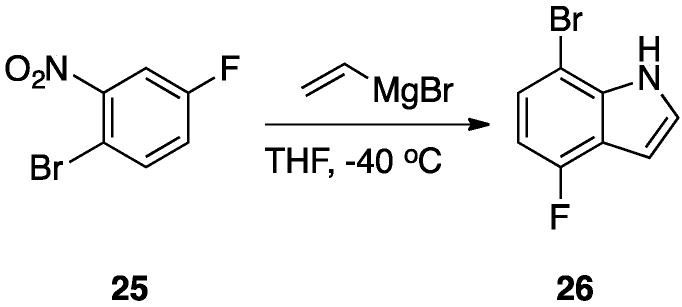



A solution of 1-bromo-4-fluoro-2-nitrobenzene **25** (10 g, 45.5 mmol) in THF (200 mL) was added dropwise to a solution of 1 M vinylmagnesium bromide in THF (182 mL, 182 mmol) at −40 °C (bath temp). The reaction was stirred at −40 °C for 3 h, and saturated aqueous NH_4_Cl was added. The layers were separated, and the organic layer was evaporated. The crude product was purified flash chromatography, giving 4.2 g (43%) of 26 (43% yield). LC-MS (ESI): m/z [M + 1]^+^ = 215.15.

##### Preparation of 4-Fluoro-1*H*-indole-7-carbonitrile (**27**)

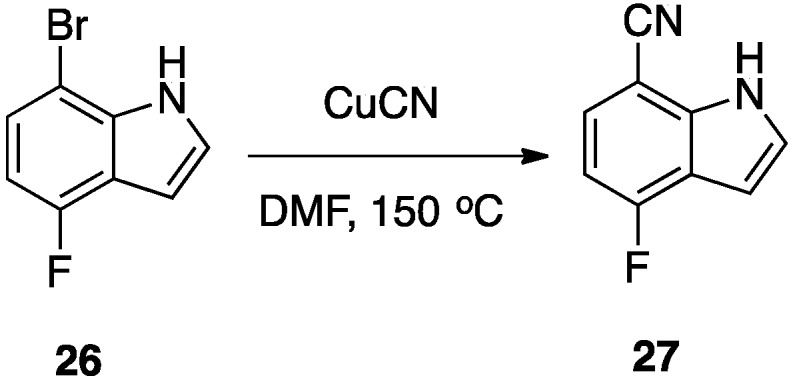



A mixture of **26** (2.2 g, 10.3 mmol) and CuCN (4.6 g, 51.4 mmol) in DMF (20 mL) was refluxed for 16 h. After cooling to room temperature, the reaction mixture was poured onto a solution of ammonia in MeOH (100 mL, sat.) and the solid was removed by filtration. The filtrate was added to a mixture of water (50 mL)/ammonia (60 mL, sat. aq.) and extracted with EtOAc/Ether (1/1) until TLC analysis showed no product in the aqueous phase. The combined organic extracts were washed with brine (2 × 200 mL) and water (200 mL), dried (Na_2_SO_4_); concentrated under reduced pressure. The residue was purified by silica gel column chromatography (petroleum spirit/ethyl acetate = 3:1, *v*/*v*) to give 4-fluoro-7-cyanoindole **27** as a tan yellow solid, yield 55%), LC-MS (ESI): *m/z* [M + 1]^+^ = 161.23.

##### Preparation of Methyl 2-(7-cyano-4-fluoro-1*H*-indol-3-yl)-2-oxoacetate (**28**)

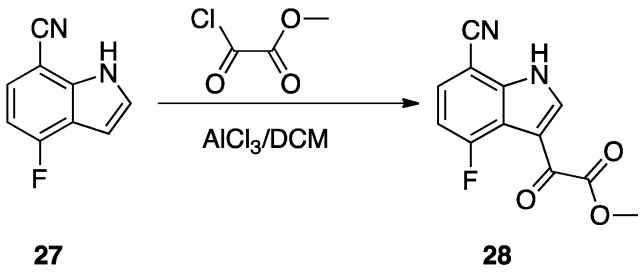



To a mixture of 4-fluoro-1H-indole-7-carbonitrile (900 mg, 5.6 mmol) in DCM (15 mL) was added methyl-2-chloro-2-oxoacetate (2.84 g, 8.76 mmol), then the reaction mixture was cooled to 0 °C, AlCl_3_ (1.5 g, 11.2 mmol) was added in portions, then the reaction mixture was stirred at 0 °C for 30 min.Then reaction mixture was quenched with MeOH and extracted with ethyl acetate (3 × 30 mL) and washed with brine. The organic layer was dried over anhydrous Na_2_SO_4_, filtered and concentrated under reduced pressure. The residue was purified by silica gel column chromatography (petroleum spirit/ethyl acetate = 3:1, *v*/*v*) to give methyl 2-(7-cyano-4-fluoro-1*H*-indol-3-yl)-2-oxoacetate (660 mg, 48% yield). LC-MS (ESI): *m*/*z* [M + 1]^+^ = 247.44.

##### Preparation of 2-(7-Cyano-4-fluoro-1*H*-indol-3-yl)-2-oxoacetic Acid (**29**)

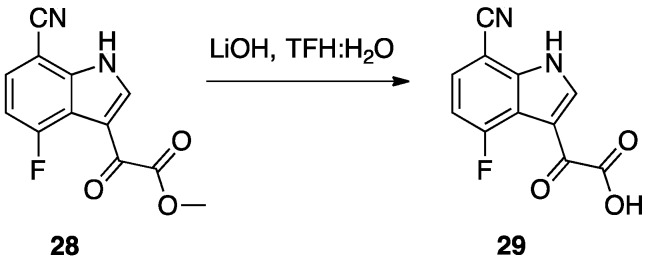



To a mixture of methyl 2-(7-cyano-4-fluoro-1*H*-indol-3-yl)-2-oxoacetate (**28**, 660 mg, 2.68 mmol) in THF (10 mL) and water (2.5 mL), LiOH (257.4 mg, 10.7 mmol) was added, this mixture was stirred at room temperature for 4 h, the reaction was monitored as by LC-MS, the solvent was concentrated and acidified with 2N HCl, extracted with ethyl acetate (25 mL × 2).The combined organic layers were washed with brine and dried over Na_2_SO_4_,the mixture was concentrated to provide 2-(7-cyano-4-fluoro-1*H*-indol-3-yl)-2-oxoacetic acid (**29**, 560 mg, 90% yield) as a yellow solid. LC-MS (ESI): *m*/*z* [M + 1]^+^ = 233.28.

##### Preparation of *N*-(3-benzoyl-3-azabicyclo[3.1.0]hexan-6-yl)-2-(7-cyano-4-fluoro-1*H*-indol-3-yl)-2-oxoacetamide (**30**)

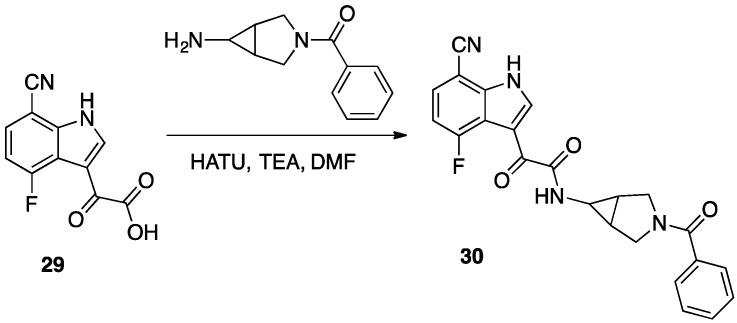



At rt, to a mixture of 2-(7-cyano-4-fluoro-1*H*-indol-3-yl)-2-oxoacetic acid (**29**, 560 mg, 2.41 mmol) in DMF (5 mL) was added (6-amino-3-azabicyclo[3.1.0]hexan-3-yl)(phenyl)methanone (488.2 mg, 2.41 mmol), HATU (1.37 g, 3.6 mmol) and TEA (731.0 mg, 7.23 mmol), then the reaction mixture was stirred at RT for 2 h. Then reaction mixture was filtered through Celite. The filtrate was concentrated under reduced pressure. The residue was purified by silica gel column chromatography (petroleum ether/ethyl acetate = 3:1, *v*/*v*) to give **30** (534 mg, 53% yield). LC-MS (ESI): *m*/*z* [M + 1]^+^ = 417.51.

##### Preparation of *N*-(3-benzoyl-3-azabicyclo[3.1.0]hexan-6-yl)-2-(4-fluoro-7-(*N*-hydroxy-carbamimidoyl)-1*H*-indol-3-yl)-2-oxoacetamide (**31**).

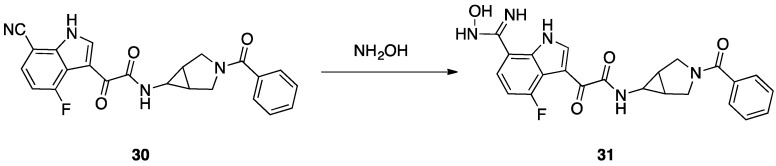



At rt, to a mixture of **30** (534 mg, 1.28 mmol) in EtOH (8 mL) was added hydroxylamine hydrochloride (178.4 mg, 2.56 mmol), and TEA (388.56 mg, 3.84 mmol), then the reaction mixture was stirred at RT for 3 h. Then reaction mixture was diluted with ethyl acetate (30 mL) and washed with brine. The organic layer was dried over anhydrous Na_2_SO_4_, filtered and concentrated under reduced pressure. The residue was purified by silica gel column chromatography (petroleum ether/ethyl acetate = 3:1, *v*/*v*) to give **31** (315 mg, 55% yield). LC-MS (ESI): *m*/*z* [M + 1]^+^ = 450.63.

##### Preparation of *N*-(3-benzoyl-3-azabicyclo[3.1.0]hexan-6-yl)-2-(4-fluoro-7-(5-(trichloro-methyl)-1,2,4-oxadiazol-3-yl)-1*H*-indol-3-yl)-2-oxoacetamide (**32**)

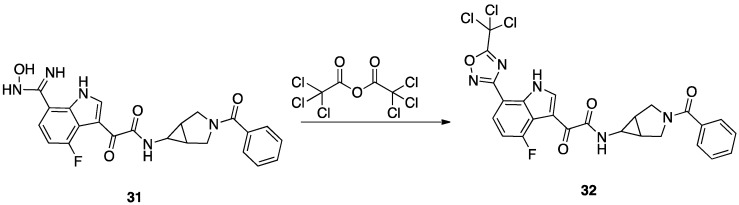



To a mixture of **31** (315 mg, 0.7 mmol) in 2,2,2-trichloroacetic anhydride (3 mL) then the reaction mixture was stirred at 80 °C for 10 h. The reaction mixture was cooled to rt, petroleum ether (10 mL) was added and the mixture was filtered to give **32** (61 mg, 15% yield) as a brown solid. LC-MS (ESI): *m/z* [M + 1]^+^ = 577.82.

##### Preparation of 2-(7-Nitro-1*H*-indazol-3-yl)acetic acid **SC55**

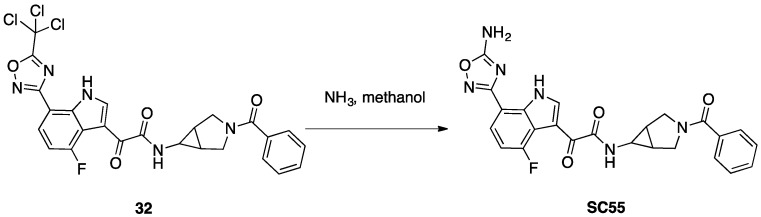



To a solution of **32** (61 mg, 0.11 mmol) in DMF (1 mL) was added a solution of ammonia in MeOH (7 M, 2 mL) and the resulting mixture stirred at r.t. for 16 h. The reaction mixture was concentrated, the residue was purified by prep. HPLC (C18, 0~90 acetonitrile in H_2_O with 0.1% formic acid) to provide **SC55** (6.1 mg, 12.2% yield) as a white solid. LC-MS (ESI): *m/z* [M + 1]^+^ = 475.41, ^1^H-NMR (400 MHz, CDCl_3_): δ 10.59 (s, 1H), 9.07 (d, *J* = 3.2 Hz, 1H), 7.87 (dd, *J* = 8.3,4.4 Hz, 1H), 7.60–7.47 (m, 1H), 7.42–7.26 (m, 4H), 7.00 (dd, *J* = 10.4,8.4 Hz, 1H), 5.45 (s, 2H), 4.28 (d, *J* = 12.7 Hz, 1H), 3.74–3.50 (m, 2H)2.60 (s, 1H), 2.21–1.91 (m, 1H), 1.89–1.73 (m, 2H).

#### 3.2.5. Synthesis of **SC56**

##### Preparation of 7-Bromo-4-methoxy-1*H*-indole (**34**)

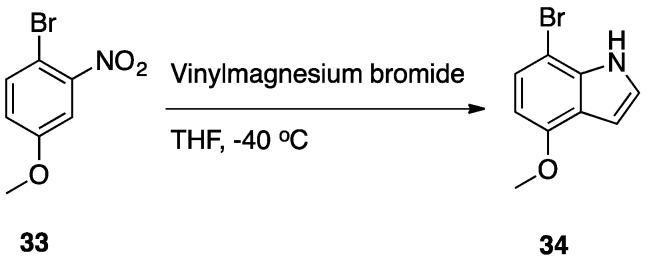



To a solution of 1-bromo-4-methoxy-2-nitrobenzene (33, 10.0 g, 43.1 mmol) in THF (100 mL) were added vinylmagnesium bromide (110 mL) dropwise at −40 °C. The reaction mixture was sealed and stirred at −40 °C for 1.5 h. After warmed up to room temperature, the resulting solution was quenched with sat. NH_4_Cl (120 mL) and extracted with ethyl acetate (2 × 150 mL). The combined organic layers were washed with brine (50 mL) and H_2_O (50 mL), dried over anhydrous Na_2_SO_4_, filtered and concentrated under reduced pressure. The residue was purified by silica gel column (petroleum ether/ethyl acetate = 2:1, *v*/*v*) to give **34** as a yellow solid (6.8 g, yield = 70.1%). LC-MS (ESI): *m*/*z* [M/M + 1]^+^ = 226.04/228.04.

##### Preparation of 4-Methoxy-1*H*-indole-7-carbonitrile (**35**)

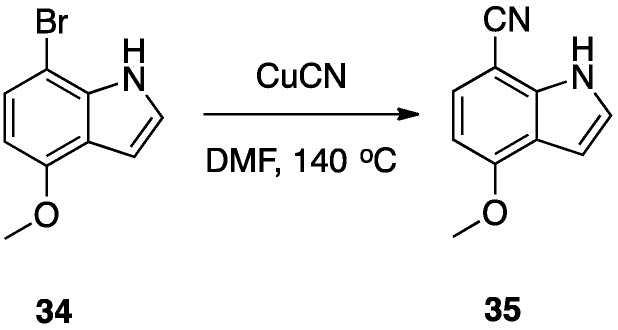



An oven-dried screw cap test tube was charged with a magnetic stir bar, CuCN (152.4 mg, 0.8 mmol), then **34** (6.8 g, Y = 70.1%) in DMF (75 mL) was added into the tube. The reaction mixture was stirred at 140 °C overnight. After cooled down to room temperature, the resulting solution was filtered through Celite. The filtrate was diluted with ethyl acetate (100 mL) and washed with saturated NaHCO_3_ solution (30 mL), water (30 mL) and brine (30 mL). The organic phase was dried over anhydrous Na_2_SO_4_, filtered and concentrated under reduced pressure. The residue was purified by silica gel column (petroleum ether/ethyl acetate = 1:1, *v*/*v*) to give **35** as a yellow solid (3.4 g, yield = 65.8%). LC-MS (ESI): *m*/*z* [M + 1]^+^ = 173.10.

##### Preparation of Methyl 2-(7-cyano-4-methoxy-1*H*-indol-3-yl)-2-oxoacetate (**36**)

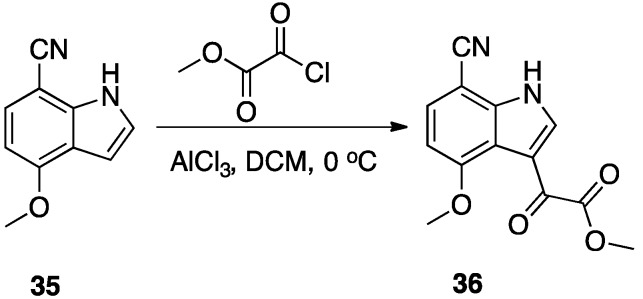



To a mixture of **35** (3.4 g, 19.8 mmol) and AlCl_3_ (13.2 g, 98.8 mmol) in DCM (80 mL) was added methyl oxalyl chloride (3.6 g, 19.7 mmol) in DCM (30 mL) slowly at 0 °C. The reaction mixture was stirred for 30 h and then quenched with saturated NaHCO_3_ solution (10 mL), and extracted with DCM (30 mL). The organic phase was washed with brine (10 mL) and H_2_O (10 mL), dried over anhydrous Na_2_SO_4_, filtered and concentrated under reduced pressure. The residue was purified by silica gel column (petroleum ether/ethyl acetate = 1:1, *v*/*v*) to give **36** as a yellow solid (2.2 g, Y = 43.1%). LC-MS (ESI): *m*/*z* [M + 1]^+^ = 259.09.

##### Preparation of 2-(7-Cyano-4-methoxy-1*H*-indol-3-yl)-2-oxoacetic Acid (**37**)

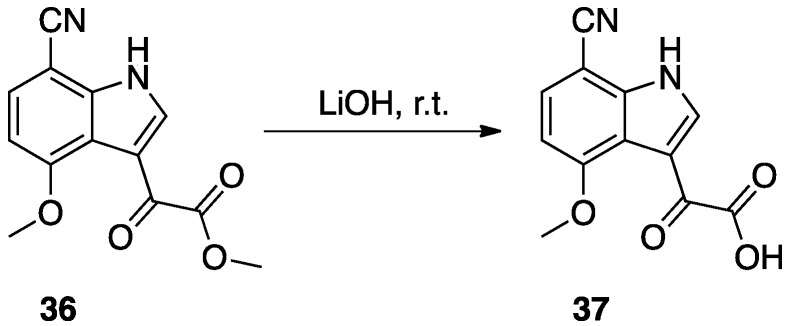



A mixture of **36** (2.2 g, 8.5 mmol) and LiOH (306.0 mg, 12.8 mmol) in THF/H_2_O (18 mL, 5:1, *v*/*v*) was stirred at room temperature for 2.5 hr. The reaction mixture was adjusted to pH = 6 with 1 N HCl (12.8 mL) and extracted with DCM (20 mL). The organic phase was concentrated under reduced pressure to give **37** as a white solid (630 mg, yield = 30.3%). LC-MS (ESI): *m*/*z* [M + 1]^+^ = 245.09.

##### Preparation of *N*-(3-Benzoyl-3-azabicyclo[3.1.0]hexan-6-yl)-2-(7-cyano-4-methoxy-*1H*-indol-3-yl)-2-oxoacetamide (**38**)

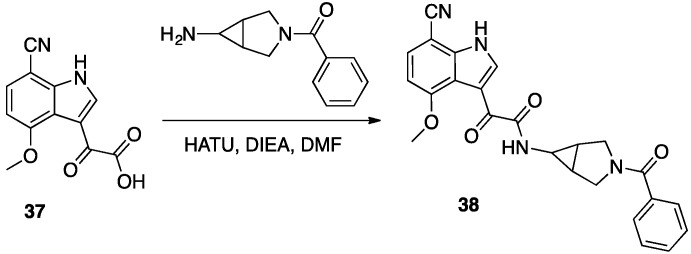



At room temperature, to a stirred solution of **37** (500 mg, 2.05 mmol) in DCM (30 mL) were added (6-amino-3-azabicyclo[3.1.0]hexan-3-yl)(phenyl)methanone HCl salt (540 mg, 2.26 mmol) and DIPEA (1 mL, 6.15 mmol), followed by HATU (935 mg, 2.46 mmol) in portions. The resulting mixture was stirred at room temperature for 30 min, and then diluted with DCM (10 mL) and water (20 mL). The organic layer was washed with brine, dried over Na_2_SO_4_, filtered and concentrated under reduced pressure. The residue was purified by flash chromatography (silica gel, 0~20% MeOH in DCM) to afford the title compound **38** (300 mg, yield = 34%). LC-MS (ESI): *m*/*z* [M + 1]^+^ = 429.21.

##### Preparation of *N*-(3-benzoyl-3-azabicyclo[3.1.0]hexan-6-yl)-2-(7-(*N*-hydroxycarbamimidoyl)-4-methoxy-1*H*-indol-3-yl)-2-oxoacetamide (**39**)

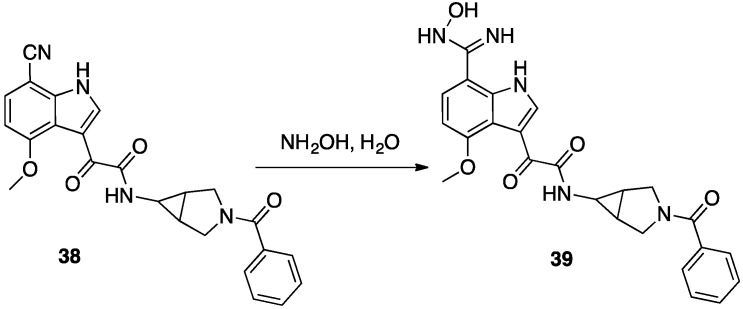



At room temperature, to a solution of **38** (300 mg, 0.7 mmol) in EtOH (10 mL) was added NH_2_OH (3 mL, 50 wt.% in water). After stirred at room temperature for 30 min, the reaction mixture was partitioned between DCM and water. The organic layer was separated, and the aqueous layer was extracted with DCM. The combined organic layers were dried over Na_2_SO_4_, filtered and concentrated under reduced pressure to give the crude title product **39**, which was used in the next step without further purification (323.0 mg, yield = 100.0%). LC-MS (ESI): *m*/*z* [M + 1]^+^ = 462.17.

##### Preparation of *N*-(3-benzoyl-3-azabicyclo[3.1.0]hexan-6-yl)-2-(4-methoxy-7-(5-(trichloro-methyl)-1,2,4-oxadiazol-3-yl)-1*H*-indol-3-yl)-2-oxoacetamide (**40**)

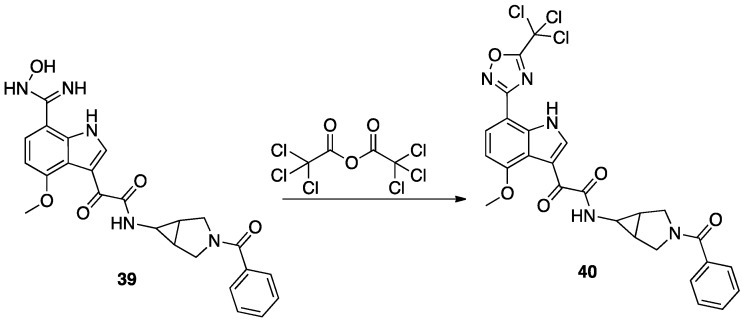



At room temperature, to a solution of **39** (323.0 mg, 0.7 mmol) in anhydrous THF (10 mL) was added 2,2,2-trichloroacetic anhydride (648.0 mg, 2.1 mmol) dropwise. The resulting mixture was stirred at room temperature overnight. After completion of the reaction as indicated by LC-MS, the reaction was quenched with ice water. The resulting solution was extracted with DCM. The combined organic layers were washed with water, dried over Na_2_SO_4_, filtered and concentrated under reduced pressure to give the crude title product **40**, which was used in the next step without further purification. LC-MS (ESI): *m*/*z* [M/M + 1]^+^ = 588.00/590.00.

##### Preparation of 2-(7-(5-Amino-1,2,4-oxadiazol-3-yl)-4-methoxy-1*H*-indol-3-yl)-*N*-(3-benzoyl-3-azabicyclo[3.1.0]hexan-6-yl)-2-oxoacetamide (**SC56**)

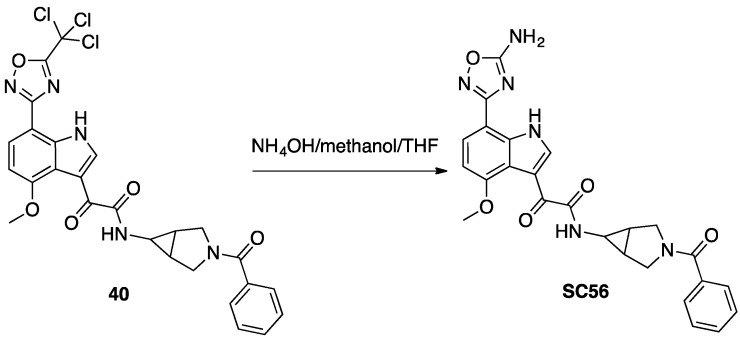



At room temperature, to a solution of **40** (411.0 mg, 0.7 mmol) in THF (10 mL) was added ammonia solution (5 mL). After stirred at room temperature for 1 h, the reaction mixture was partioned between DCM and water. The organic layer was separated, and the aqueous layer was extracted with DCM. The combined organic layers were washed water, dried over Na_2_SO_4_, filtered and concentrated under reduced pressure. The residue was purified by prep-HPLC (Gilson, C18, 10%~100% MeCN in water with 0.1% formic acid) to afford **SC56** (4.3 mg, yield = 1%) as a white solid. LC-MS (ESI): *m/z* [M + 1]^+^ = 487.10; ^1^H-NMR (400 MHz, CDCl_3_): *δ* 10.55 (s, 1H), 8.94 (d, *J* = 3.2 Hz, 1H), 7.93 (d, *J* = 8.4 Hz, 1H), 7.56 (br. s, 1H), 7.46–7.38 (m, 5H), 6.81 (d, *J* = 8.4 Hz, 1H), 5.38 (s, 2H), 4.36–4.32 (m, 1H), 4.04 (s, 3H), 3.75-3.66 (m, 3H), 2.64 (s, 1H), 1.91-1.83 (m, 2H).

### 3.3. Cells

HEK293T cells (a gift from Dr. Irwin Chaiken, Drexel University, Philadelphia, PA, USA) were cultured in Dulbecco’s Modified Eagle’s Medium (DMEM), 10% FBS, 100 U/mL penicillin, 100 µg/mL streptomycin and 2mM l-glutamine. Human astroglioma U87 cells stably expressing CD4/CCR5 or CD4/CXCR4 (obtained from Prof. Hongkui Deng, Peking University, China and Prof. Dan Littman, New York University, New Yory, NY, USA, through the AIDS Research and Reference Reagent Program, Division of AIDS, NIAID, NIH) [40,41] were cultured in DMEM supplemented with 10% FBS, 100 U/mL penicillin, 100 µg/mL streptomycin and 2 mM l-glutamine, 300 µg/mL G418 (Thermo Scientific, Waltham, MA, USA) and 1µg/mL puromycin (Thermo Scientific, Waltham, MA, USA). Cells were incubated continuously in a humidified 5% CO_2_/95% air environment at 37 °C.

### 3.4. Proteins

HEK293F cells were used to express the soluble cleaved trimer B41 SOSIP.664 gp140. The recombinant trimer was subsequently purified by mAb 2G12-affinity chromatography followed by size-exclusion chromatography as described previously (PMID: 25589637); IgG b12 anti HIV-1 gp120 was obtained through the NIH AIDS Reagent Program, Division of AIDS, NIAID, NIH: Anti-HIV-1 gp120 Monoclonal (IgG1 b12) from Dr. Dennis Burton and Carlos Barbas); p24 was produced in-house as previously described [42]. Briefly, a vector containing C-terminally His-tagged HIV-1_NL4-3_CA (a gift from Dr. Eric Barklis, Oregon Health and Science University, Portland, OR, USA) was transformed into BL21-Codon Plus (DE3)-RIL Competent Cells (Agilent Technologies, Wilmington, DE, USA) and expressed in autoinduction ZYP-5052 medium at 30 °C while shaking at 250 rpm overnight [43]. Bacterial cultures were spun down at 8000 rpm, and the supernatant was discarded. Cell pellets were resuspended in 1× PBS and lysed via sonication. The lysed sample was spun down at 45,000 rpm, the clarified supernatant was filtered through a 0.45 µm filter, and immediately applied to a Talon cobalt resin affinity column (Clonetech Laboratories, Mountain View, CA, USA). Bound protein was eluted using 1× PBS with 250 mM imidazole. Elutions containing purified CA-H6 were pooled, dialyzed overnight into 20 mM Tris-HCl pH 8.0 at 4 °C, concentrated to 120 µM, flash frozen, and stored at −80 °C.

### 3.5. Production of Pseudotyped Viruses

Single-round infectious envelope-pseudotyped luciferase-reporter viruses were produced by a co-transfection of two vectors (3:4 ratio of vector 1:2) in 6-well plated 293T cells (1 × 10^6^ cells/well) [41]. Vector 1 is an envelope-deficient HIV-1 pNL4-3-Luc+R-E plasmid which carries the luciferase-reporter gene [33]. Vector 2 is a plasmid expressing the HIV-1 gp160 Env from the various isolates tested (B41, Hxbc2, YU2, JRCSF, and JRFL) [26,35,36,37,38,39,44,45,46]. Transient transfections of these vectors were carried out via calcium phosphate (ProFection Mammalian Transfection System, Promega, Madison, WI, USA) for 5 h. The DNA-containing medium was replaced with fresh culture media after the 5 h transfection incubation. Supernatants containing pseudovirus were collected 72 h post transfection, clarified, filtered, aliquoted and stored at −80 °C.

### 3.6. ELISA-Based Quantification of p24 Content

ELISA plate was coated with 50 ng/well of mouse anti-p24 (ab9071, Abcam, Cambridge, MA, USA) overnight at 4 °C. Following the overnight incubation, the plate was blocked with 3% (*w*/*v*) BSA at room temperature for 2 h and washed with 0.5% (*v*/*v*) Tween in PBS. Pseudoviral stocks were lysed using 0.1% (*v*/*v*) Triton X-100 (Sigma-Aldrich, St. Louis, MO, USA) at 37 °C for 1 h and added to the plate overnight at 4 °C. Simultaneously, p24 protein (produced and purified as previously described) was used as a standard. The following day, the plate was washed with 0.5% PBST and a 1:5000 dilution of rabbit anti-p24 (ab63913, Abcam) was added for 2 h at room temperature. After washing with PBST to remove the unbound rabbit anti-p24 off the plate, goat anti-rabbit-HRP at a 1:5000 dilution was added for 1 h at room temperature. The plate was then extensively washed with PBST. Subsequently, a solution of 0.4 mg/mL *O*-phenylenediamine in a phosphate-citrate buffer with sodium perborate (Sigma-Aldrich) was added and incubated for 30 min in the dark. Optical densities were then obtained at 450 nm in a Multiskan™ GO Microplate Spectrophotometer (Thermo Scientific).

### 3.7. Single-Round Infection Assay

The single-round HIV-1 infection assay was performed as previously described [33,47,48]. Briefly, U87.CD4.CCR5/CXCR4 (1.2 × 10^4^ cells/well) target cells were seeded in 96-well luminometer-compatible tissue culture plates (Greiner Bio-one, Monroe, NC, USA). After 24 h, compound or DMSO (vehicle control for compounds, Sigma) were mixed with pseudotyped viruses (normalized to p24 content), added to the target cells, and incubated for 48 h at 37 °C. Following the 48 h incubation, the media was removed from each well, and the cells were lysed using 50 μL/well of 1X luciferase lysis buffer (Promega) and one freeze-thaw cycle. After adding 50 µL/well of luciferase assay substrate (Promega), a GloMax 96 microplate luminometer (Promega) was used to measure the luciferase activity of each well.

### 3.8. Cellular Toxicity

The viability of the U87.CD4.CCR5/CXCR4 cells was determined using the Cell Counting Kit-8 cell proliferation and cytotoxicity assay (Dojindo Molecular Technologies, Rockville, MD, USA) per the manufacturer’s instructions. U87.CD4.CCR5/CXCR4 cells (1.2 × 10^4^ cells/well) were seeded in 96-well tissue culture plates (Olympus Plastics, San Diego, CA, USA). After 24 h, cells were treated with compound (0.1–1000 µM) or DMSO (Sigma) for 48 h at 37 °C. Subsequently, 10 µL of the CCK-8 solution was added to each well and incubated for 4 h at 37 °C. Following the 4 h incubation, the absorbance at 450 nm was measured in a Multiskan™ GO Microplate Spectrophotometer (Thermo Scientific). Untreated cells were used as a background control and 0.1% SDS treated cells were used as a positive control. The 50% cytotoxicity (CC_50_) value was defined as the concentration of the compounds that reduced the viability (observed as a decrease in absorbance) of treated cells by 50% as compared with control cells.

### 3.9. SPR Direct Interaction Analysis

#### Immobilization of Env Constructs

Interaction analyses were performed on a ProteOn XPR36 SPR Protein Interaction Array System (Bio-Rad Laboratories, Hercules, CA, USA) at 25 °C for all kinetic analyses. ProteOn GLH sensor chips were preconditioned with two short pulses each (10 s) of 50 mM NaOH, 100 mM HCl, and 0.5% (*w*/*v*) sodium dodecyl sulfide. Then the system was equilibrated with PBST buffer (20 mM Na-phosphate, 150 mM NaCl, and 0.005% [*v*/*v*] polysorbate 20, pH 7.4). The surface of a GLH sensorchip was activated by a 10 min injection with a 1:100 dilution of a 1:1 mixture of 1-ethyl-3-(3-dimethylaminopropyl)carbodiimide hydrochloride (0.2 M) and sulfo-*N*-hydroxysuccinimide (0.05 M). Immediately after chip activation, 100 µg mL^−1^ soluble cleaved gp140 B41.SOSIP.664 trimers in 10 mM sodium acetate, pH 5.0 was injected across ligand flow channels for 15 min at a flow rate of 25 µL min^−1^. A 5-min injection of 1 M ethanolamine HCl (pH 8.5) was then performed to cap excess active ester groups on the sensor surface.This resulted in the immobilization of Env constructs at a density of 14,000 RUs (response unit, which is an arbitrary unit that corresponds to 1 pg/mm^2^). A reference surface was similarly created by immobilizing a non-specific protein (IgG b12) in 10 mM sodium acetate, pH 5.0 to match the density of immobilized HIV-1 proteins.

### 3.10. Direct Binding Analysis

To prepare the compounds for analysis, the compound stock solutions were brought up to 30 μL in 100% DMSO and this was made to a final volume of 1 mL by addition of sample preparation buffer (PBS, pH 7.4) to ensure that the concentration of DMSO was matched with that of running buffer (3% DMSO). Serial dilutions from a starting concentrating as indicated in the result section were then prepared in running buffer (PBS, 3% [*v*/*v*] DMSO, 0.005% [*v*/*v*] polysorbate 20, pH 7.4) and injected across the surfaces at a flow rate of 100 μL min^−1^, for a 2.6 min association phase, followed by up to a 10 min dissociation phase using the “one shot kinetics” capability of the Proteon instrument [49]. Data were analyzed using the ProteOn Manager Software version 3.0 (Bio-Rad). To account for nonspecific binding and injection artifacts, the responses of a buffer injection and responses from the reference flow cell were subtracted. Experimental data were fitted to a simple 1:1 binding model (where applied). The average kinetic parameters (association [k_a_] and dissociation [k_d_] rates) generated from five data sets were used to define the equilibrium dissociation constant (K_D_).

### 3.11. Negative Stain Electron Microscopy (EM)

SC compounds were dissolved in 100% methanol to a final concentration of 0.2 mg/mL and 10 μL aliquots (total of 2 μg compound) were evaporated using a SpeedVac. 400 μL of B41 SOSIP.664 (at 0.25 mg/mL in Tris-buffered saline [TBS]) were added to tubes containing lyophilized compound. The small molecules were resuspended by pipetting up and down and left to incubate at room temperature for 1 h. Final concentrations of trimer (approximate MW of peptide 215,000 Da) and small molecule (MW 513.5 Da) were estimated to be 0.93 μM and 9.73 μM, respectively. A control tube containing only B41 SOSIP.664 (at 0.25 mg/mL Tris-buffered saline) was also incubated under the same conditions. Following incubation, each sample was diluted to about 0.02 mg/mL protein concentration in TBS and a 3 μL aliquot was adsorbed onto a glow-discharged, carbon-coated Cu400 copper mesh grid. The drop was blotted using Whatman #1 filter paper, a 3 μL drop of 2% (*w*/*v*) uranyl formate was added to the sample-treated surface of the grid, and the sample stained for 45 s before blotting. Samples were imaged using an FEI Talos Arctica electron microscope (Thermo Fisher) operating at 200 kV and an FEI Ceta 16M CMOS camera (Thermo Fisher). Single frame exposures were collected with a total dose of ~25 e^−^/Å^2^ at a magnification of 73,000, resulting in a pixel size of 1.98 Å at the specimen plane. Data processing and analysis has been described previously [26,50].

### 3.12. Differential Scanning Aalorimetry (DSC)

Experiments were performed using a MicroCal VP-Capillary differential scanning calorimeter (Malvern Panalytical, Westborough, MA, USA). Before the experiments were carried out, B41 SOSIP.664 was dialyzed against phosphate-buffered saline (PBS). The protein concentration was subsequently adjusted to 0.25 mg/mL. Similar to the EM methods above, 0.5 mL of B41 SOSIP.664 were added to tubes containing 2 μg lyophilized compound **SC11**, **SC15**, **SC28** or **SC45**, mixed by pipetting, and allowed to incubate for 1 h at room temperature. The samples, along with a control containing protein alone, were into the instrument cell and thermal denaturation was probed at a scan rate of 90 °C/h, with PBS in the reference cell. Buffer correction, normalization, and baseline subtraction procedures were performed using the Automated Origin 7.0 software (Malvern Panalytical, Westborough, MA, USA). The data were fitted using a non-two-state model.

### 3.13. Molecular Modeling

#### 3.13.1. Docking of Compounds **SC11**, **SC15** and **SC45**

The Env protein (pdb code: 6MUG) was prepared by the Protein Preparation Wizard implemented with Maestro (Schrödinger Maestro Version 11.5.011, New York, NY, USA, MM share Version 4.1.011, New York, NY, USA, Release 2018-1, Platform Darwin-x86_64). The Grid box was centered on the co-crystalized BMS-386150. For validating the glide docking protocol, the original ligand BMS-386150 was built using the LigPrep tool (Schrödinger Maestro Version 11.5.011, New York, NY, USA) and docked using glide-XP mode. The predicted docked pose matched the original co-ordinates of the co-crystalized BMS-386150 with an RMS value of 0.1 Å. **SC11**, **SC15** and **SC45** was docked using the same glide-XP mode and the top ranked pose was selected. The docked protein-ligand complexes were then refined using Prime (VSGB solvation model and OPLS3e forcefield, entire protein refinement).

#### 3.13.2. Docking of Compounds **SC28**, **SC49**, **SC50**, **SC52**, **SC55** and **SC56**

Glide induced-Fit (extended sampling settings) was used to flexibly sample the ligands and the protein pocket. The top ranked poses were then refined using Prime (VSGB solvation model and OPLS3e forcefield, entire protein refinement).

## 4. Conclusions

In this study, we demonstrated that four entry inhibitors with different core scaffolds still interact with the B41 SOSIP.664 gp140 trimer via SPR but with different kinetics. After examining the effects of these four compounds on the overall structure of B41 SOSIP.664 trimer using NS-EM and DSC, we discovered that all of them, including **SC28** (azabicyclohexane core), were able to stabilize the SOSIP and the EM results suggested a shift in the conformational equilibrium of the SOSIP from a heterogeneous population of open and closed trimers to a more homogenous pool of closed trimers. The findings suggest that a molecule like **SC28** is able to stabilize the SOSIP and halt or severly impair the ability of receptor-mediated conformational dynamics of Env, making it a potential allosteric fusion inhibitor. Using computational field- and structure-based design methods, we chose five analogues of **SC28** that had a modified methyltriazole-azaindole head group and retained the azabicyclo-hexane core region. These five **SC28** derivatives all retained target specificity to B41 SOSIP.664 gp140 trimers and exhibited antiviral activity. Most striking was the difference between **SC28** and **SC56**, both compounds had nearly identical potencies, but had a 1000-fold difference in affinity. We have previously demonstrated for this class of compounds that the dissociation rate is correlated to the potency and when looking at the contributing factor to this increase in affinity, we observed that both compounds had similar dissociation rates, but that the association rates also differed by a factor of 1000. This further confirms that by decreasing the dissociation rate we can improve the potency for future analogues of our entry inhibitors to help bring this class of inhibitors closer towards clinical utility. By continuing to explore compounds that shift the conformational equilibrium of Env trimers, this class of entry inhibitors will have profound implications for future inhibitor and vaccine design efforts.

## Figures and Tables

**Table 1:** Kinetics and affinity of compounds **SC11**, **SC15**, **SC28**, and **SC45** binding to B41 SOSIP.664 gp140 trimer.

Compound	k_a_ (M^−1^s^−1^)	k_d_ (s^−1^)	K_D_
**SC11**	3.83 ± 1.12 × 10^3^	5.02 ± 2.67 × 10^−4^	0.131 µM
**SC15**	3.01 ± 0.188 × 10^5^	5.44 ± 0.677 × 10^−3^	0.0181 µM
**SC28**	1.39 ± 0.14 × 10^4^	6.99 ± 0.43 × 10^−3^	0.511 µM
**SC45**	1.38 ± 0.045 × 10^4^	1.52 ± 0.0294 × 10^−2^	1.10 µM

**Table 2:** Calculated Overall Ligand-Protein interaction energies for the novel azabicyclohexane analogues.

Compound	Calculated Overall Ligand-Protein Interaction Energy Kcal/Mol
**SC28**	−16.13700
**SC49**	−6.58188
**SC50**	−12.87666
**SC52**	−2.25288
**SC55**	−10.12901
**SC56**	−18.37654

**Table 3:** Kinetics and affinity of novel **SC28** derivatives binding to soluble, cleaved recombinant Env. N.D. = not determined.

Compound	k_a_ (M^−1^s^−1^)	k_d_ (s^−1^)	K_D_ (µM)
**SC28**	1.39 ± 0.14 × 10^4^	6.99 ± 0.43 × 10^−3^	0.504 µM
**SC49**	9.49 ± 1.6 × 10^2^	3.43 ± 0.36 × 10^−2^	36.1 µM
**SC50**	N.D.	N.D.	114 ± 51 µM
**SC52**	N.D.	N.D.	23.7 ± 2.4 µM
**SC55**	N.D.	N.D.	11.4 ± 3.7 µM
**SC56**	1.22 ± 0.067 × 10^7^	6.39 ± 0.31 × 10^−3^	0.526 nM

**Table 4:** Potency and toxicity of compounds against HIV-1_B41_, HIV-1_HxBc2_, HIV-1_JRCSF,_ HIV-1_JRFL,_ and HIV-1_YU-2_ Env pseudotyped HIV-1. The chemical structures were drawn with ChemAxon software (Budapest, Hungary).

Compound	IC_50_ B41 (µM)	IC_50_ HxBc2 (µM)	IC_50_ JRCSF (µM)	IC_50_ JRFL (µM)	IC_50_ YU-2 (µM)	Median IC_50_ (µM)	CC_50_ (µM)	Therapeutic Index (CC_50_/IC_50_)
**SC28** (parental) 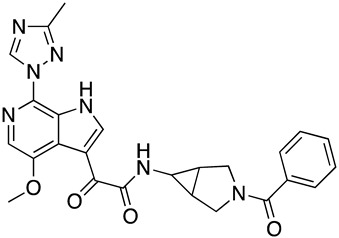	0.035 ± 0.0062	0.021 ± 0.005	0.026 ± 0.009	0.17 ± 0.05	0.48 ± 0.05	0.15 ± 0.17	190 ± 54	1291.21
**SC49** 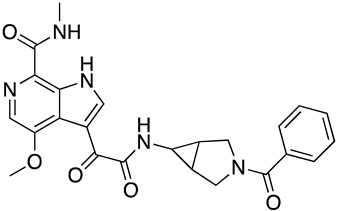	26 ± 19	62 ± 1.4	NA	33.32 ± 11.56	57.2 ± 26.5	45 ± 15	326 ± 81	7.31
**SC50** 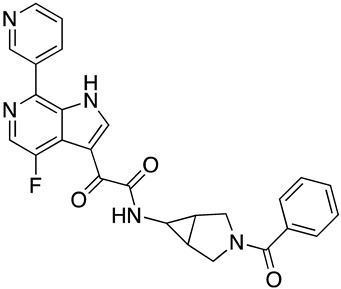	7.1 ± 4.2	6.9 ± 5.2	28.92 ± 5.62	24.28 ± 6.2	22.6 ± 4.2	18 ± 9	120 ± 18	6.63
**SC52** 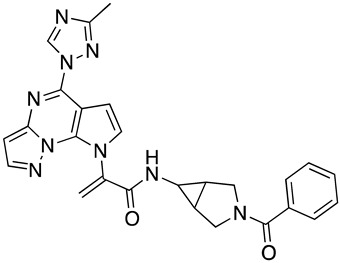	0.43 ± 0.1	0.44 ± 0.17	2.02 ± 0.25	NA	1.35 ± 0.44	1.06 ± 0.668	9.2 ± 1.1	8.65
**SC55** 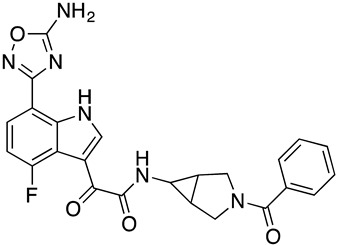	2.02 ± 0.81	0.73 ± 0.56	0.45 ± 0.35	0.45 ± 0.33	1.15 ± 0.81	0.96 ± 0.59	73 ± 14	75.85
**SC56** 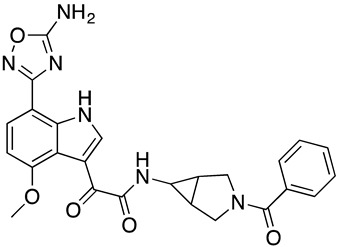	0.051 ± 0.008	0.011 ± 0.003	0.046 ± 0.009	0.13 ± 0.015	0.11 ± 0.049	0.0698 ± 0.045	94 ± 10	1344.71

## Supplementary Materials

The following are available online at, Figure S1: Single round infection assay graphs, Figure S2: Toxicity of compounds, Figure S3: Sensorgram of **SC56**, Table S1: NS-EM statistics.
