# Supplementary material for: Field-Based Affinity Optimization of a Novel Azabicyclohexane Scaffold HIV-1 Entry Inhibitor
Source: Molecules. 2019 Apr 22;24(8):1581. doi: 10.3390/molecules24081581 (PMC6514670; doi:10.3390/molecules24081581)
Supplement: Supplementary file 1 [file molecules-24-01581-s001.pdf]

Supplementary Materials

Field-based Affinity Optimization of a Novel Azabicyclohexane Scaffold HIV-1 Entry Inhibitor

Megan E. Meuser [1], Adel A. Rashad [1], Gabriel Ozorowski [2], Alexej Dick [1], Andrew B. Ward [2] and Simon Cocklin [1,*]

[1] Department of Biochemistry & Molecular Biology, Drexel University College of Medicine, Rooms 10307, 10309, and 10315, 245 North 15th Street, Philadelphia, PA 1910, USA; mem484@drexel.edu (M.E.M.); aaa396@drexel.edu (A.A.R.); ad3474@drexel.edu (A.D.)

[2] Department of Integrative Structural and Computational Biology, Collaboration for AIDS Vaccine Dis covery, The Scripps Research Institute, La Jolla, CA 92037, USA; gozorows@scripps.edu (G.O.); andrew@scripps.edu (A.B.W.)

* Correspondence: sc349@drexel.edu; Tel.: 215-762-7234 (Office); 215-762-4979 (Lab); Fax: 215-762-4452

Commented [m1]: Please carefully check the accuracy of names and affiliations. Changes will not be possible after proofreading.

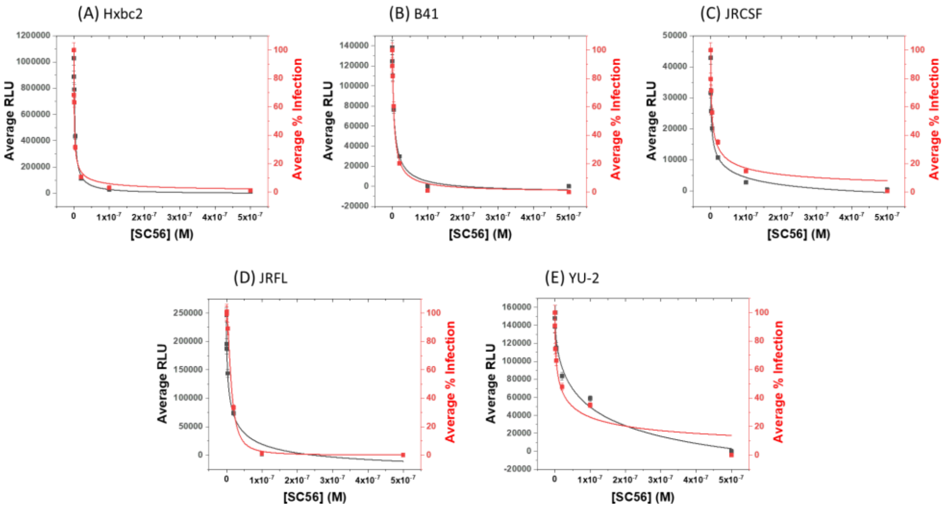

Figure S1. Single round infection assay graphs, using **SC56** (starting concentration 5μM with 1:5 serial dilutions) as an example, against pseudoviruses with envelopes derived from (A) HxBc2 (B) B41 (C) JRCSF (D) JRFL and (E) YU-2. The left axis (black) represents the average relative luciferase units while the right axis (red) represents the average % infection. A non-linear regression logistic fit of data points from which the IC50 values were derived is shown. Error bars indicate the standard deviation from at least three individual replicates.

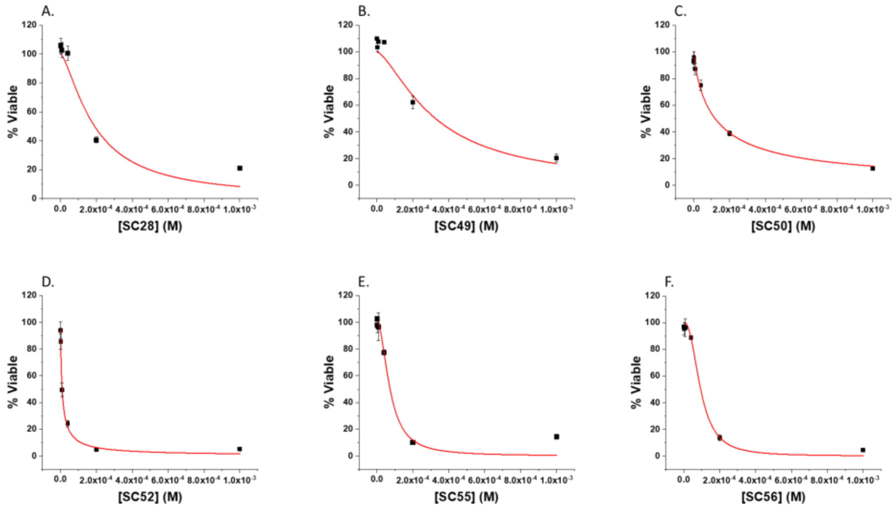

Figure S2. Toxicity of compounds (A) **SC28**, (B) **SC49**, (C) **SC50**, (D) **SC52**, (E) **SC55**, and (F) **SC56** starting at 1mM with 1:10 dilutions using U87 cells. Red line represents the logistic fit of the data points from which the CC_{50} was derived. Error bars indicate the standard deviation from three individual replicates.

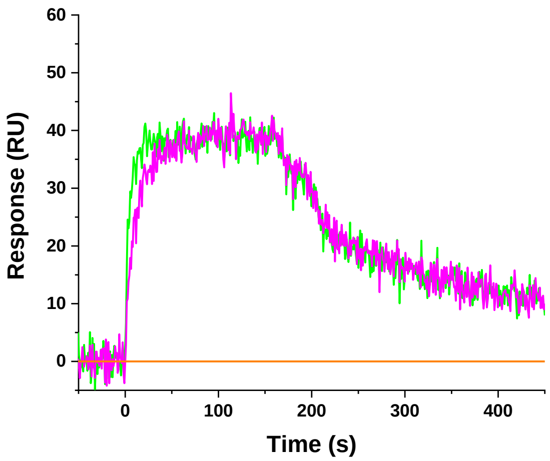

Figure S3. Sensorgrams depicting 12.5 μM (green) and 6.25 μM (magenta) **SC56** over a surface to which 13000 RUs of B41 SOSIP.664 gp140 trimer had been immobilized. The theoretical Rmax for this surface is 41 RUs. As can be seen, both concentrations saturate the surface.
